# A biohybrid mesh harvester for distributed energy harvesting in living tissues

**DOI:** 10.1126/sciadv.aei5963

**Published:** 2026-10-07

**Authors:** Siqi Wang, Xiaoyu Wang, Shuai Fu, Claire Senger, Jacob Pfund, Menka Jain, Reika Katsumata, Jun Yao

**Affiliations:** ^1^ Department of Electrical and Computer Engineering, University of Massachusetts, Amherst, MA, USA.; ^2^ Department of Polymer Science and Engineering, University of Massachusetts, Amherst, MA, USA.; ^3^ Department of Physics, University of Connecticut, Storrs, CT, USA.; ^4^ Institute for Applied Life Sciences, University of Massachusetts, Amherst, MA, USA.; ^5^ Department of Biomedical Engineering, University of Massachusetts, Amherst, MA, USA.

## Abstract

Harnessing bio-origin resources for energy conversion offers a promising pathway toward energy sustainability matched to biosystems, addressing a fundamental limitation in powering bio-integrated electronics. However, existing approaches by integrating harvesters onto continuous substrates follow a “centralized” paradigm that differs markedly from the inherently distributed energy conversion found in living tissues, limiting both biocompatibility and scalability. We demonstrate a distributed harvesting strategy by integrating individual harvesting units within a mesh network that closely mimics the structural and mechanical properties of biological tissues. This architecture enables seamless embedding within in vitro cardiac tissue to form a bio-hybridization with cellular-scale intimacy across both device and substrate, transcending conventional approaches limited to a surface contact. This strategy achieves effective energy density more than an order of magnitude higher than that of existing approaches based on the centralized paradigm. While the concept is currently demonstrated in an in vitro cardiac system for converting biomechanical energy, the approach can provide a generalizable framework for integrating diverse energy-harvesting modalities and offers a pathway toward in vivo biohybrid systems.

## INTRODUCTION

Engineering bio-integrated electronics, represented by various wearable and implantable devices, has enabled improved health monitoring, therapeutic intervention, and performance assistance (*1*–*5*). Continuous efforts are devoted to mitigating the mismatches, evident in physical and chemical properties (*5*–*7*), between electronic and biological systems to enhance the interfacing intimacy. Beyond this, biosystems still differ from conventional electronic systems in their ability to sustain their own energy while maintaining high mobility. Improving energy sustainability in bioelectronics is therefore essential for sustaining their bio-integrated functions, yet it remains a considerable challenge. Batteries, as the predominant energy source (*8*, *9*), encounter limitations, because efforts to engineer their form factors (e.g., flexibility, miniaturization) for improved bio-integration intimacy often come at the cost of reduced energy volume (*10*). These constraints underscore the need for alternative powering strategies to achieve improved energy sustainability. Among them, the concept of converting on-site biomechanical, biochemical, and biothermal energy has received considerable attention (*11*–*13*), as leveraging these bio-origin sources may fundamentally reduce the mismatch in energy sustainability between electronic and biological systems.

Biomechanical energy, as a representative example, is associated with a wide spectrum of activities ranging from body motions to internal physiological processes, and thus represents a promising source for continuous energy supply to bio-integrated devices. To this end, harvesters based on piezoelectric and triboelectric effects have been developed (*14*). A common strategy is to integrate these harvesters on flexible substrates that can conformally adhere to cutaneous surfaces. Motion-induced curvature changes are transmitted through the flexible substrates to the harvesters for energy conversion. This approach has also been extended to internal organs to power implantable devices, with additional packaging, surgical, and suturing procedures required to ensure biosafety and biostability. In these cases, harvester-integrated flexible patches are either sutured onto organ surfaces (e.g., epicardium) or inserted in muscle tissue to capture contractile motion for energy conversion (*15*–*20*). Other strategies include the deployment of harvesters within internal chamber spaces (e.g., the ventricle or vasculature) to exploit biomechanical energy from fluidic motion (*21*, *22*), which reduce the need for direct tissue adhesion and potentially lower the risk of inflammation.

Collectively, these approaches can be categorized into a ‘centralized’ harvesting paradigm, in which harvesting units are packaged together as an ensemble. The packaging not only introduces potential invasiveness to the biosystem but also reduces the effective energy conversion density (e.g., sub-μW/cm^3^ in existing piezoelectric implants) (*15*, *17*). Moreover, this paradigm remains fundamentally distinct from energy supply in biosystems, where energy conversion is distributed throughout the tissue volume (e.g., ATP conversion at the cellular level) for broad accessibility and high efficiency. In the context of signal communication, it has long been recognized that emulating the distributed nature of biological signaling is critical for advancing bio-integration; accordingly, recent developments have produced filamentary, tissue-like sensing networks for bio-integration (*6*, *23*–*30*). By analogy, we envision that establishing a similarly distributed energy-harvesting network could enable truly biohybrid powering and fundamentally enhance the sustainability of bio-integrated systems.

In this regard, integrating individual harvesting units directly within tissue represents a logical step toward true bio-hybridization. Previous efforts have demonstrated that embedding arrays of piezoelectric microcantilevers in cell cultures can solicit energy signals from the cellular motions (*31*). However, these harvesting units are integrated on a rigid planar substrate, suggesting that the entire system still behaves like a centralized, bulky device incapable of genuine bio-hybridization. These limitations highlight the need to re-engineer all peripheral elements such as substrate and interconnects in the system to achieve full compatibility with biological systems, including minimized feature sizes and tissue-matched mechanical properties.

## RESULTS

### Conceptualization and design

Our strategy for engineering a biohybrid energy harvesting system centers on both structural and functional considerations. Specifically, the architecture interconnecting individual energy harvesting units is equally important for establishing a distributed system that minimizes perturbation to native tissue function. Recent advances in mesh electronics (*24*–*29*), consisting of interconnected ribbon networks with cellular-scale feature sizes and tissue-like mechanical compliance, offer a promising platform for distributed, minimally invasive tissue interfacing. Our approach is to leverage such a mesh architecture by integrating thin-film harvesters within the network to enable the formation of a truly distributed harvesting system.

Without losing generality, we intended to integrate the bioelectronic mesh harvester with in vitro cardiac tissues to evaluate the bio-integration and harvesting strategy. Specifically, cardiac cells will be seeded onto the released mesh harvester, which can serve as a scaffold for continuous cell growth and tissue development, owing to its porous structure that resembles the extracellular matrix (*23*) (Fig. 1A). During the subsequent culture period, the growing cardiomyocytes are expected to progressively reinforce connection across the interstitial mesh spacing and form a continuous laminar microtissue, which naturally integrates the mesh. The aligned, filamentary mesh ribbons can also promote cellular alignment, guiding cardiac contraction along the longitudinal axis (Fig. 1B). Correspondingly, the energy harvesting units integrated on the mesh are oriented along these ribbons to efficiently capture strain induced during cardiac contraction for energy conversion. As the filamentary structure in the mesh ribbons can promote cell attachment for forming a stable tissue integration, it is expected that tissue deformation can be effectively transduced into bending of the mesh scaffold. The contraction-relaxation cycles in the cardiac tissue impose cyclic strain in the harvesting units, providing a suitable mechanical stimulus for piezoelectric energy conversion.

**Fig. 1.**
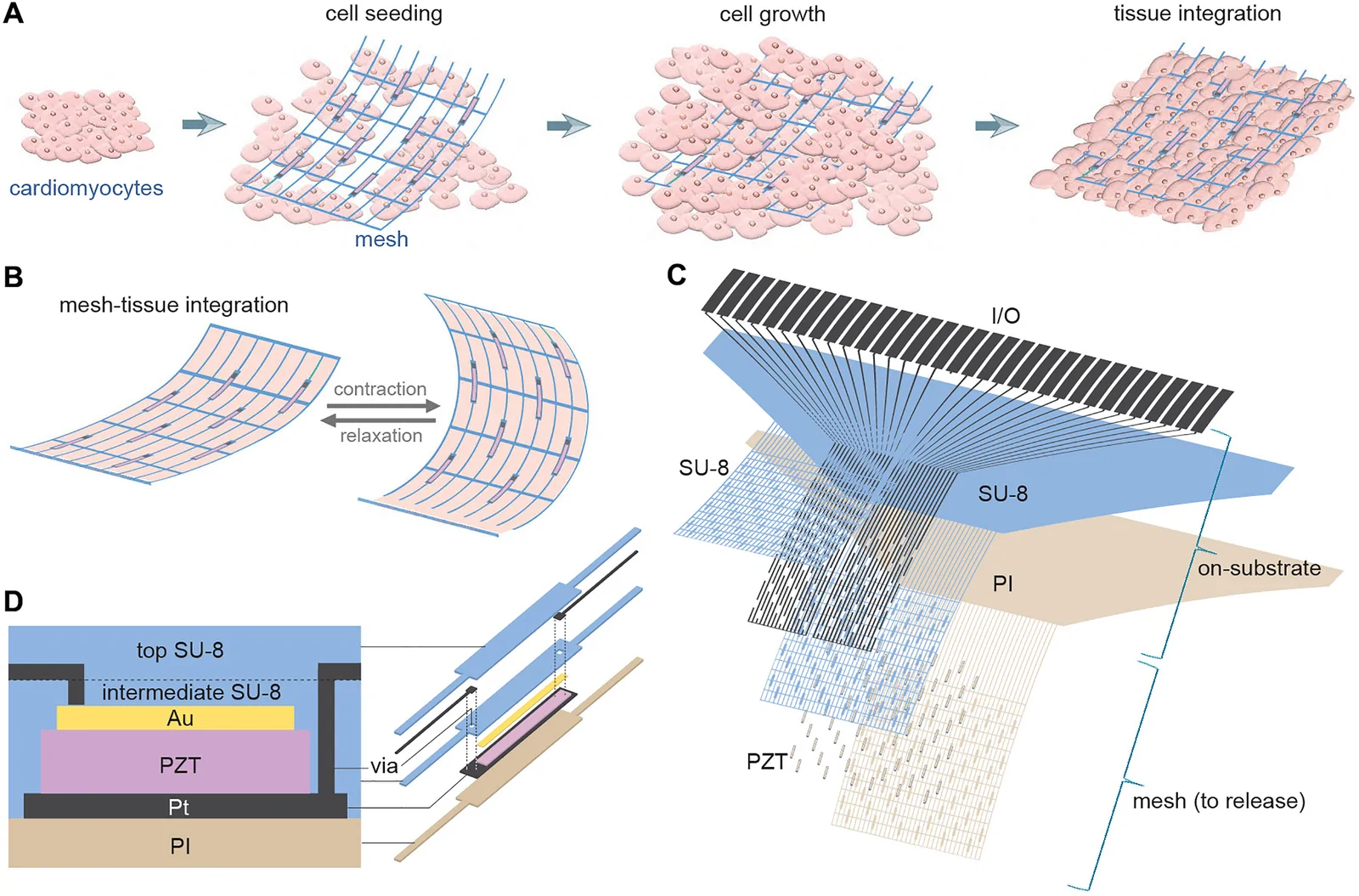
Biohybrid mesh harvester concept and design. (**A**) Schematics of forming biohybrid mesh-tissue integration. (**B**) Schematic showing mechanical transduction from tissue contraction to integrated harvesting units. (**C**) Exploded-view schematic of the mesh harvester system. (**D**) Schematic of the multilayer device structure in the harvesting unit.

The proposed mesh harvester system comprises two parts (Fig. 1C). The first is a mesh network integrated with harvesting units and will be eventually released from the substrate for cell seeding and tissue integration. The second on-substrate component includes the extending interconnects and terminal input/output (IO) contacts that connect to an external measurement system. The mesh network consists of ribbons, which have lateral dimensions smaller than a typical cell size (e.g., 20–100 μm) to promote cell attachment and an ultrathin thickness to ensure mechanical flexibility. These ribbons are interconnected to form rectangular lattice, resulting in a highly porous architecture. The lattice spacing is considerably larger than a cell size to facilitate cell infiltration. Each ribbon features a multilayer structure, in which the interconnect (Pt) is sandwiched between bottom and top dielectric layers that provide mechanical support and electrical insulation. In this design, polyimide (PI) is selected as the bottom dielectric layer for its high mechanical resilience (e.g., fracture limit ∼80%) (*32*) to enhance structural integrity. SU-8 is used as the top dielectric layer, leveraging its convenience for multilayer lithographic patterning. Both materials are biocompatible and broadly used in bioelectronic tissue interfaces (*25*, *32*). The piezoelectric harvesting unit consists of a thin film of lead zirconate titanate (PbZr_0.52_Ti_0.48_O_3_, or PZT) sandwiched between a pair of electrodes. It is patterned into a slender rectangular geometry aligned with the ribbons to facilitate bending and support cell attachment (Fig. 1D). Electrical access to the PZT unit is achieved through via-like openings defined in an intermediate SU-8 layer to establish connections to the top and bottom electrodes. A final top SU-8 capping layer is deposited to complete the electrical insulation of both the PZT units and interconnects.

### Device fabrication

The overall system design was subsequently translated into device fabrication. A PZT thin film (∼350 nm thick) was first deposited by a sol-gel process (*33*) onto a SiO_2_/Si substrate pre-coated with a 300 nm thick Pt layer (fig. S1). The underlying Pt layer can facilitate the formation of well-defined crystalline domains in the PZT, which is beneficial for its piezoelectric response (*34*). X-ray diffraction (XRD) analysis revealed a dominant (111) polycrystalline orientation of PZT (fig. S2), consistent with prior reports (*35*). The (111) orientation of PZT facilitates electromechanical coupling by enabling strain components along multiple directions to contribute to the electrical response, and has been shown to enhance the piezoelectric properties of PZT (*36*). To define the PZT harvesting units, photolithography was employed to define a sacrificial mask layer on top of the PZT layer, followed by reactive ion etching (RIE) to define PZT stripes (Fig. 2Aa). A second similar selective etching was used to define the Pt pattern, exposing the surrounding SiO_2_ layer of the substrate. Notice that the underlying Pt layer also served as the bottom electrode, and was therefore patterned with a larger footprint extending beyond the PZT region to enable electrical addressing.

**Fig. 2.**
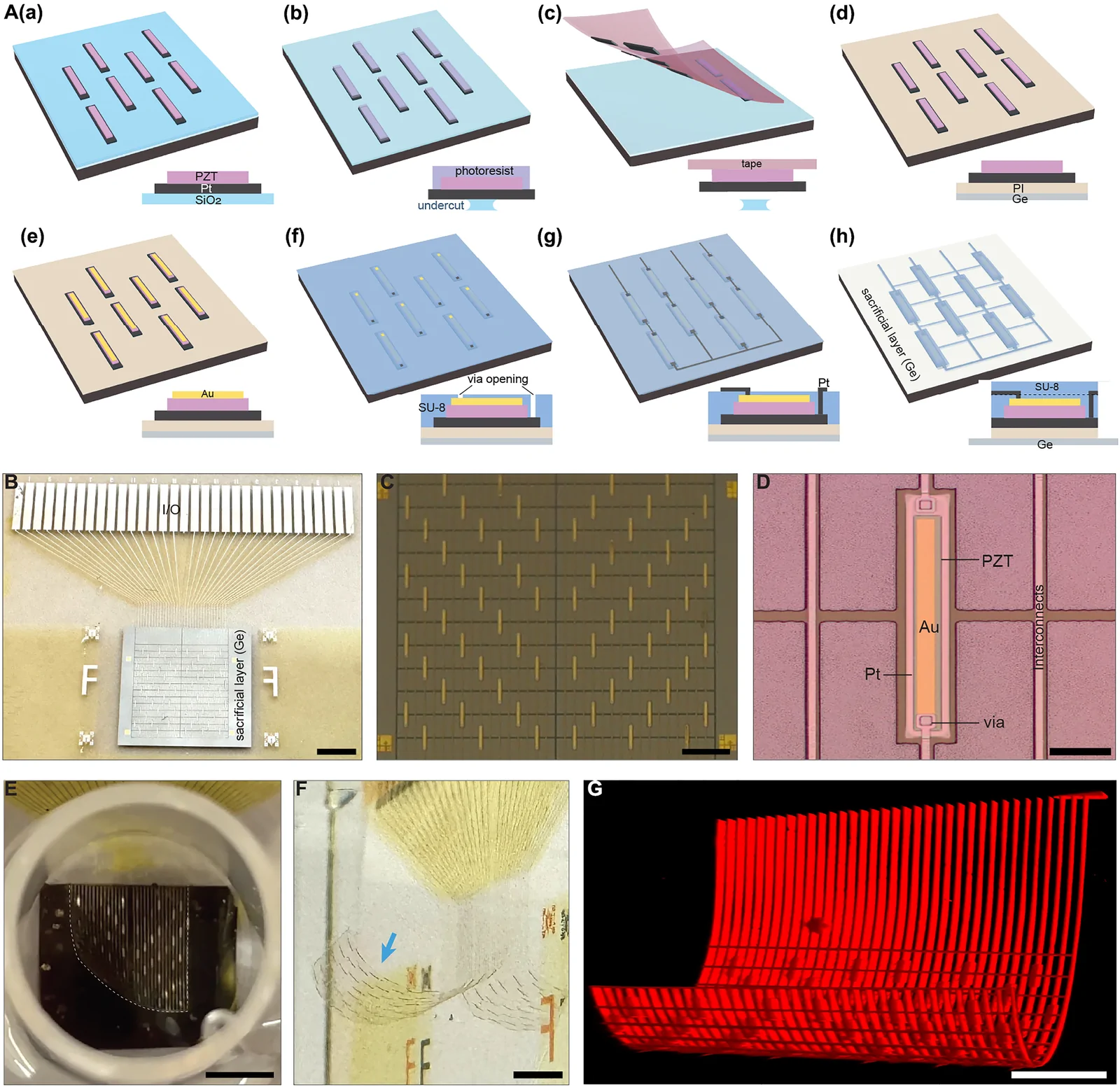
Fabrication of the mesh harvester system. (**A**) Schematics of the fabrication flow. (**B**) A fabricated mesh system before releasing from the substrate. Scale bar, 2.5 mm. (**C**) Integrated 16 × 4 PZT unit array. Scale bars, 0.5 mm. (**D**) A zoom-in optical image of the PZT unit. Scale bar, 100 μm. (**E**) A fabricated mesh released from the substrate. Scale bar, 3.5 mm. (**F**) Optical image of a released mesh floating in water, forming bending structure (arrow). Scale bar, 2 mm. (**G**) Fluorescence image of a released mesh in water, bending along its longitudinal direction. Scale bar, 1.5 mm.

We then employed a peel-and-transfer method (*37*, *38*) to transfer the patterned PZT/Pt units onto a PI layer for subsequent mesh definition and integration. Briefly, the defined PZT/Pt units were first protected by patterning a resist layer (Fig. 2Ab), followed by immersion in hydrofluoric acid (HF). The HF selectively etched the exposed SiO_2_ surrounding the structures, generating lateral undercut beneath the PZT/Pt units and thereby weakening their adhesion to the substrate. A thermal release tape was used to peel the PZT/Pt units from the substrate (Fig. 2Ac) and transfer them onto a PI layer (∼1.5 μm thick) coated on a Ge-coated Si substrate (Fig. 2Ad). In this configuration, the PI layer served as the bottom dielectric of the mesh, whereas the Ge layer functioned as a sacrificial layer for the eventual release of the mesh system (*28*). It is noteworthy that we developed a strategy to improve material utilization and fabrication efficiency, in which PZT/Pt units can be selectively peeled off and transferred (fig. S3). This approach enables the definition of dense PZT/Pt unit arrays and supports multiple transfer cycles from a single source substrate for scalable device fabrication. Following transfer, a top Au electrode was defined on the PZT units using standard photolithography, metal deposition, and lift-off processes (Fig. 2Ae). A SU-8 layer was then spin-coated and lithographically defined with via-like openings for electrical contacts to both the top (Au) and bottom (Pt) electrodes of the PZT unit (Fig. 2Af). Electrical contacts and interconnects (Pt) were subsequently defined by standard lithographic steps. A second SU-8 layer (∼2 μm thick) was coated to encapsulate the PZT units and interconnects (Fig. 2Ag). Finally, a resist sacrificial mask defining the mesh geometry was patterned, and RIE was employed to selectively remove the dielectric layers, yielding the final mesh architecture (Fig. 2Ah).

The mesh system covered an area of 7 × 8 mm^2^ (Fig. 2B), comparable to the typical dimensions of laminar microtissues (*39*). A 16 × 4 array of PZT units was integrated on the mesh (Fig. 2C), with each size ∼50 × 470 μm^2^ (Fig. 2D). Along the longitudinal direction, four adjacent units were connected in series as a harvesting device. The mesh ribbons had a lateral width of 20 μm and total thickness ∼3.7 μm, yielding an estimated bending stiffness ∼3.42 × 10^−13^ N·m^2^ that was comparable to previously reported mesh systems designed for tissue embedment. The lattice spacings between neighboring ribbons along the longitudinal and lateral directions were 180 and 480 μm, respectively, generating a porosity ∼78% in the system. The mesh was released from the substrate by etching the Ge sacrificial layer (Fig. 2E). Once released, the mesh readily underwent bending in aqueous solution (Fig. 2F), indicating a high degree of mechanical compliance consistent with the designed structural parameters. Notably, the design promoted preferential bending along the mesh’s longitudinal axis (Fig. 2G), potentially facilitating guided tissue growth and aligned contraction to enhance strain transduction to the integrated harvesting units.

### Device performance evaluations

We then carried out performance evaluations to verify device integrity and functionality prior to tissue integration. To this end, a 1 × 9 array of individual PZT units was fabricated following the same procedure described above, with each PZT unit maintaining identical structural parameters (Fig. 3A). The fabricated array was transferred onto a commercial PI film (∼125 μm thick) as the flexible substrate, which can be bended to induce strain in the PZT units for electromechanical characterizations. The bending was achieved by compressing the flexible substrate, with the PZT units positioned at the apex of the buckling region (Fig. 3B). In a representative bending, characterized by the compressing displacement (Δ*L* = 0.6 mm) against the initial substrate length (*L* = 29 mm), the nine PZT units generated an average output voltage of 0.49 ± 0.011 V (Fig. 3C), providing initial evidence of successful device fabrication with uniform performance.

**Fig. 3.**
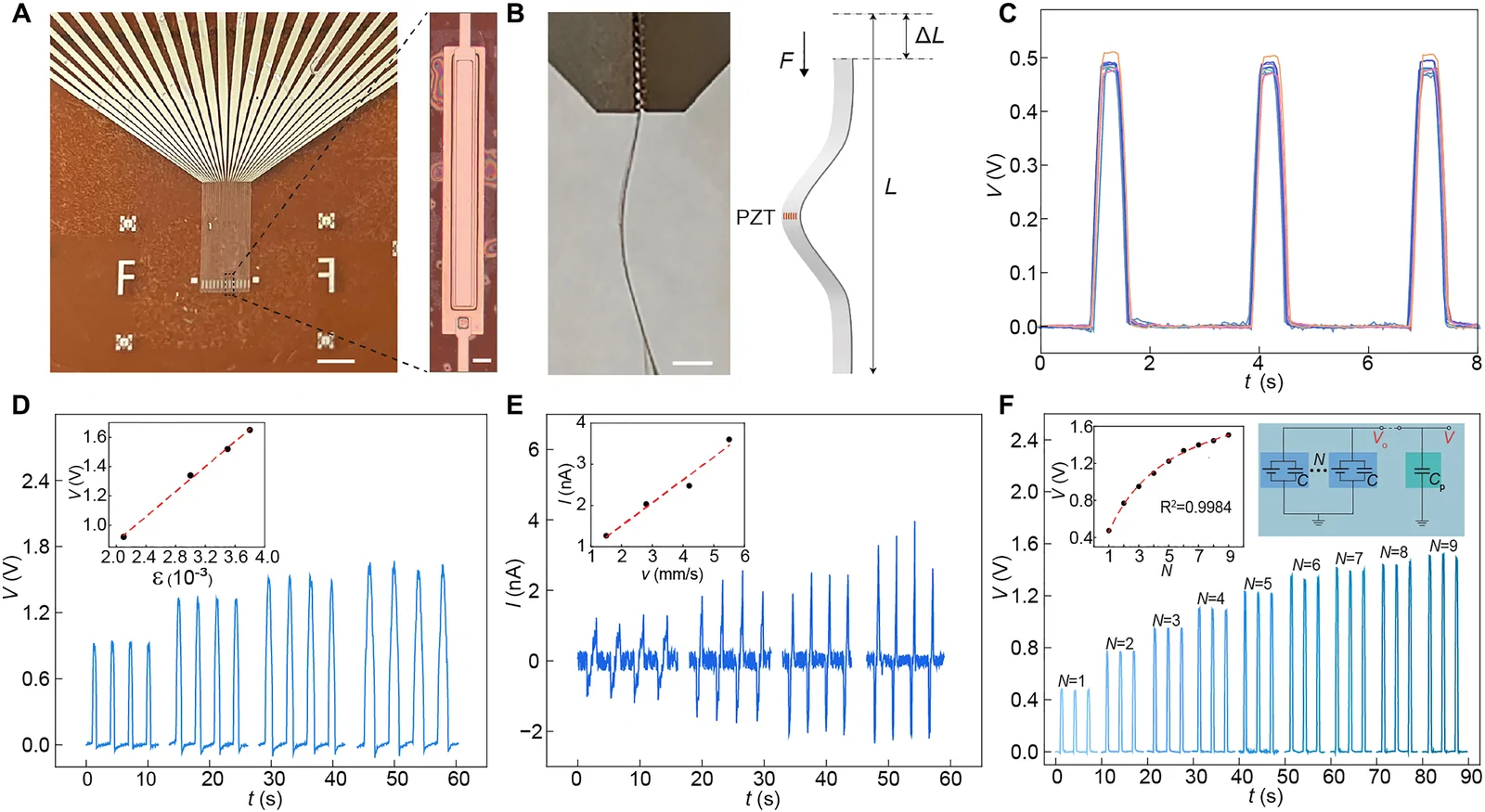
Performance evaluation of the integrated harvesting units. (**A**) Optical image of the integrated PZT units transferred onto a flexible substrate of commercial PI film. Scale bar, 2 mm. (Right) Zoom-in image of a PZT unit. Scale bar, 30 μm. (**B**) Bending the flexible substrate carrying the PZT units. Scale bar, 5 mm. (Right) Schematic illustrating the buckling bending characterized by the compressing displacement Δ*L* with respect to the substrate’s original length *L*. (**C**) Measured voltage outputs from the 9 integrated individual PZT units. (**D**) Measured voltage outputs from a PZT unit with respect to different compressing displacements Δ*L* increasing from 2.3 to 7 mm. (Inset) The relationship between the voltage output and the induced strain *ε* converted from Δ*L*. (**E**) Measured current outputs from a PZT unit with respect to compressing rates (*v* = dΔ*L*/d*t*) increasing from 1.5 to 5.5 mm/s. The current was measured by connecting the PZT unit with a load resistor of 150 MΩ. (Inset) The relationship between the current output and *v*. (**F**) Measured voltage from multiple PZT units connected in parallel. (Right inset) Schematic of the equivalent circuit, accounting for the parasitic capacitance *C*_p_ introduced by the measurement system. Each PZT unit is represented by a voltage source connected with its intrinsic capacitance *C*. The measured voltage *V* thus is related to the actual voltage output *V*_o_ from the connected PZT units as $V=V_{o}\frac{N\cdot C}{N\cdot C+C_{p}}$. (Left inset) Trend of the measured output voltages (dots) fitted by the above equation (dashed line).

For improved analysis, we referred to the constitutive equations of piezoelectricity (*40*), in which the output voltage *V* is related to the induced strain ε as (note S1)

$$V=\frac{-N\cdot \bar{e\cdot }d}{\bar{k}}e^{\frac{-N\cdot d}{A\cdot R\cdot \bar{k}}t}\int_{0}^{t}\frac{\mathrm{d\varepsilon}}{\mathrm{dt}}e^{\frac{N\cdot d}{A\cdot R\cdot \bar{k}}t}\mathrm{dt}$$ (1)

where $\bar{e}=e_{31}-\left(c_{13}/c_{33}\right)e_{33}$ and $\bar{k}=k_{33}+e_{33}^{2}/c_{33}$ represent the effective piezoelectric coefficient and dielectric constant, respectively. *d*, *A*, and *N* denote the thickness and area of the PZT layer, and the number of PZT units connected in series, respectively. *R* is the external load resistance. Given that the measurement instrument has an input resistance ∼200 TΩ, *R* can be treated as effectively infinite (i.e., open-circuit condition). In such case, Eq. 1 can be approximated as

$$V=\frac{-N\cdot \bar{e\cdot }d}{\bar{k}}\varepsilon$$ (2)

which suggests a linear relationship between the output voltage and the induced strain (note S1) (*41*).

To investigate such correlation, we increased the strain by increasing the compressing displacement Δ*L* (e.g., from 2.3 to 7 mm), which resulted in a corresponding increase in output voltages (Fig. 3D). For qualitative analysis, we sought to relate the applied displacement to the induced strain in the PZT units. Specifically, given the relatively small dimensions of the PZT units (e.g., two orders of magnitude shorter than the substrate), the substrate can be approximated as a uniform thin film, allowing its bending behavior to be analytically modeled (*42*) (note S2). The analytical model was further validated by comparison with the actual deformed geometry from optical imaging. Based on the resulting geometry and material properties, the strain profile in the PZT units was subsequently obtained. The calculated strain exhibited an approximately linear relationship with the measured output voltage (Fig. 3D, inset), consistent with the above analysis.

Meanwhile, according to the constitutive equations, the output current is proportional to the rate of strain change as $I=-A\cdot \bar{e}\cdot \frac{\mathrm{d\varepsilon}}{\mathrm{dt}}$ (note S1) (*43*). For experimental evaluation, we fixed the strain level (Δ*L* = 2.3 mm, ε = 2.1 × 10^−3^) while increasing the compressing rate ($v=\frac{d∆L}{\mathrm{dt}}$) from 1.5 to 5.5 mm/s. As expected, the measured output current increased with increasing deformation rate (Fig. 3E). Further analysis revealed an approximately linear relationship between the output current and the rate of strain change (Fig. 3E, inset), consistent with the theoretical prediction. These results further confirmed the piezoelectric mechanism and validated the functionality of the device in flexible integration.

We further investigated the performance of multiple PZT units connected in different configurations. The measured voltage output increased with the number of units connected in parallel (Fig. 3F), which is against the intuitive expectation of a constant voltage in an ideal parallel connection. Our analysis suggested that, due to the small size, the capacitance of an individual PZT unit (*C*) was relatively low and can be comparable to the parasitic capacitance (*C*_p_) introduced by the measurement system (e.g., cables and instrumentation). This could lead to a redistribution of the induced polarization charges, which can be described by an equivalent capacitive circuit model (Fig. 3F, right inset). Accordingly, the measured voltage is then reduced to $V=V_{o}\frac{N\cdot C}{N\cdot C+C_{p}}$, where *V_o_* is the actual voltage output from *N* PZT units connected in parallel. To evaluate this analysis, the capacitance of a single PZT unit was measured to be ∼100 pF (fig. S4), corresponding to an estimated relative dielectric constant of ∼480, consistent with reported values in sol-gel PZT (*44*). Using this value, the trend in the measured outputs *V* can be well described by the equivalent circuit model and accurately fitted by the above equation (Fig. 3F, left inset). The fitting further revealed a parasitic capacitance $C_{p}$ ∼ 350 pF associated with the measurement setup. This parasitic effect was further corroborated through additional measurements involving hybrid series-parallel configurations (fig. S5). Identifying and accounting such parasitic contribution is critical for accurately interpreting the electrical output of the mesh harvester system when integrated with tissues.

### Tissue integration

Following functional validation of the device, we evaluated the biosafety of the mesh for tissue interfacing. The encapsulation materials used in the mesh (a 2-μm SU-8 top layer and a 1.5-μm PI bottom layer) have been widely used and extensively validated for their biostability and biocompatibility in bioelectronic devices operating in physiological environments. Our characterizations further demonstrated that the encapsulation maintained excellent electrical insulation in cell culture medium over a period (>2 months) representative of typical in vitro cardiac tissue culture (fig. S6), while live/dead assays confirmed the biocompatibility of the mesh (fig. S7).

We then proceeded to integrate the mesh harvester system with cardiac microtissues. A cell culture chamber was mounted onto the substrate to enclose the fabricated and releasable mesh, whereas the I/O contacts remained accessible outside the chamber and were wire-bonded to a connector interfaced with the measurement system (Fig. 4A). The mesh was released from the substrate and thoroughly rinsed with deionized (DI) water. A layer of Matrigel was coated on the mesh to promote cell adhesion, as well as providing initial stabilization on the substrate. Human embryonic stem cell-derived cardiomyocytes (hESC-CMs) were then differentiated and seeded onto the mesh following established protocols (*28*, *38*). During culture, the cardiomyocytes progressively aggregated around the mesh, forming a continuous sheet or laminar cardiac microtissue (Fig. 4B). The enhancing cell-cell interactions gradually induced detachment of the tissue sheet from the substrate, initiating at the peripheral regions and propagating inward. Over time, this process led to pronounced tissue wrapping. Consistent with the mesh design, which favors bending along the longitudinal axis (Fig. 2G), the direction of tissue wrapping was preferentially aligned along this axis, potentially enhancing strain transduction to the integrated PZT devices. The coordinated deformation of the microtissue and mesh indicated a high degree of mechanical and structural integration between the two.

**Fig. 4.**
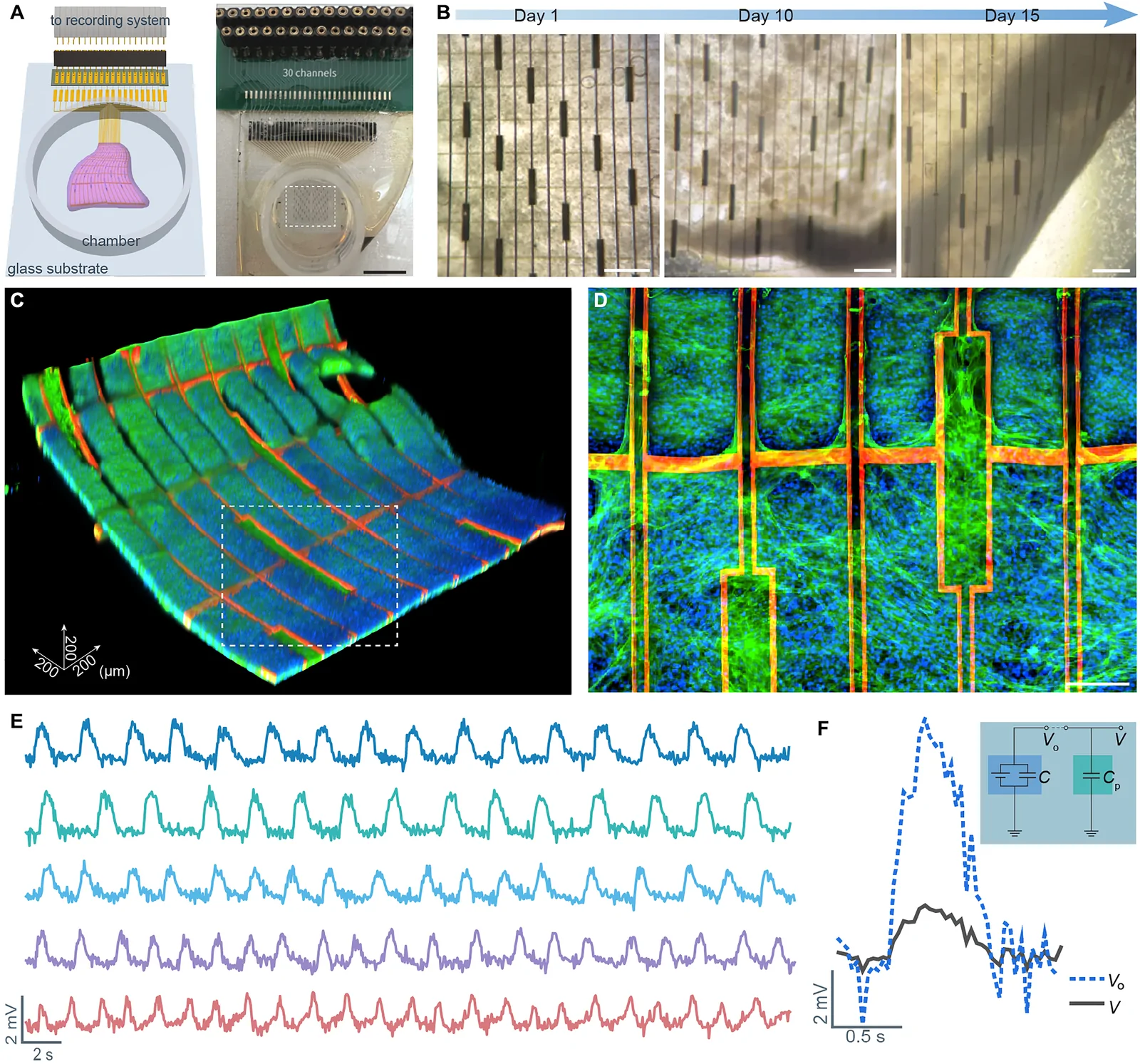
Mesh-tissue integration. (**A**) Schematic and (right) actual setup for cell seeding on the released mesh (dashed box) contained in a culture chamber, with the mesh further connected to the measurement system. Scale bar, 1 cm. (**B**) Representative images of the mesh-tissue integration process at different days after cell seeding. Scale bar, 1 mm. (**C**) Reconstructed 3D fluorescence image of the stained biohybrid mesh-tissue integration. The red, green, and blue colors correspond to the mesh, F-actin, and nuclei, respectively. (**D**) Fluorescence image of the local feature corresponding to dashed-box region in (C). Scale bar, 100 μm. (**E**) Measured voltage outputs from five representative mesh-integrated PZT devices. Note that these recordings are not synchronized, due to the limited single-channel input in the measurement system. (**F**) Calibrated actual signal amplitude $V_{o}$ relative to the measured value V, after accounting for parasitic capacitance effects introduced by the measurement system. The inset shows the equivalent measurement circuit involving the parasitic capacitance *C*_p_. The PZT device is represented by a voltage source connected with its intrinsic capacitance *C*. The measured voltage *V* thus is related to the actual voltage output *V*_o_ from the connected PZT device as $V=V_{o}\frac{C/4}{C/4+C_{p}}$, where *C* and *C*_p_ are ∼256 and 350 pF, respectively (see note S4).

Fluorescence imaging was employed to further elucidate the integration details. The imaging revealed that a substantial portion of the mesh ribbons (red) was embedded within the tissue matrix (Fig. 4C), consistent with previous observations that filamentary structures can establish highly intimate interfaces by interpenetrating cellular networks (*29*, *30*). In other word, in contrast to conventional flexible electronics that typically form only surface contacts, the mesh architecture enables volumetric embedding within the microtissue, resulting in enhanced integration and interfacial intimacy. A closing in of the interface revealed that the cytoskeletons (green) uniformly filled in the lattice spacing of the mesh, with clear attachment to ribbon structures, particularly at crossing joints (Fig. 4D). This indicates that the mesh network formed an effective tissue scaffold for cell adhesion and interconnection. Notably, dense cytoskeletal coverage was also observed over the PZT units, suggesting that their material properties and geometry support favorable cell affinity and uniform incorporation into the tissue alongside the mesh ribbons. Collectively, these observations demonstrate a seamless integration of the mesh harvester system into the cardiac microtissue, encompassing both the structural framework and functional device elements, thereby supporting the concept of bio-hybridization. More broadly, this integration provides a strategy for engineering templated laminar microtissues with potential applications in tissue transplantation (*45*), and the embedded mesh system may thus provide a pathway for transition from in vitro platforms to in vivo tissue integration.

### Bioelectrical characterizations

The intimate integration of the mesh harvester system within the laminar microtissue provides the foundation for strain transduction from tissue contraction into piezoelectric energy conversion. Electrical measurements were performed after the microtissues developed spontaneous contractions, typically at a frequency of 0.3–0.5 Hz. The measurements revealed periodic voltage signals from five representative harvesting devices, with an average signal frequency (∼0.4 Hz) matching the intrinsic contractile frequency of the microtissue (*28*, *38*) (Fig. 4E). These voltage signals exhibited an average time span ∼1.5 s, consistent with the typical timescale of the contraction phase. Notably, an asymmetry between the rising and falling edges was observed, reflecting the characteristic faster contraction and slower relaxation dynamics of cardiac tissue (*28*, *38*). This feature further suggests that the mesh can also function as a self-powered sensing system for tracking contractile dynamics. The temporal correlation and waveform characteristics indicate that the measured signals originate from cardiac contraction and are consistent with the piezoelectric transduction mechanism. Non-piezoelectric contributions such as electrochemical drifts, bioelectrical activity, and mechanical artifacts were excluded (figs. S6 and S8). The measured voltage signals had an average amplitude ∼1.4 mV. After accounting for the parasitic capacitance of the measurement system, as discussed previously (Fig. 3F), the calibrated actual voltage output was estimated to be ∼9 mV (Fig. 4F). The value, however, appeared to be considerably lower than that obtained under controlled bending conditions (Fig. 3).

We therefore further analyzed the measured signals to validate their piezoelectric origin and to assess the consistency of device performance. Given the previously established linear relationship between electrical output and induced strain (Fig. 3D), we sought to estimate the strain experienced by the PZT devices during cardiac contraction. To this end, the device was modeled as a deflected multilayer structure using classical beam theory, where the strain ε is related to the bending radius *R* as ε=∆y/R (Fig. 5Aa). Here, Δ*y* represents the distance between the mid-plane of the PZT layer $\left(y_{\mathrm{PZT}}\right)$ and the neutral plane of the entire multilayer device structure $\left(y_{\mathrm{dev}}\right)$. The bending radius *R* was estimated from the curvature of the tissue sheet by optically tracking its projection onto the substrate plane (fig. S9). Based on this analysis, the estimated strain in the PZT devices during cardiac contraction was ε ∼ 6.4 × 10^−6^ (note S3; table S1). This value was about two orders of magnitude lower than the strain (e.g., ∼10^−3^) induced during the controlled bending tests (Fig. 3D). Consistent with the linear dependence predicted by Eq. 2, this reduction in strain corresponds to a proportional decrease in output voltage, in agreement with the experimental observations. We further analyzed the intrinsic piezoelectric property of the PZT devices based on the constitutive equations and the linear approximation (Eqs. 1 and 2). Using a relative dielectric constant ($\bar{k}$ ∼480) derived from the measured capacitance of the PZT device, and a typical ratio of piezoelectric coefficients (*46*) (e_31_/e_33_ ≈ −0.6), we estimated a piezoelectric stress coefficient e_33_ ∼ 3.66 C/m^2^, falling within the expected range of sol-gel PZT materials (*47*, *48*).

**Fig. 5.**
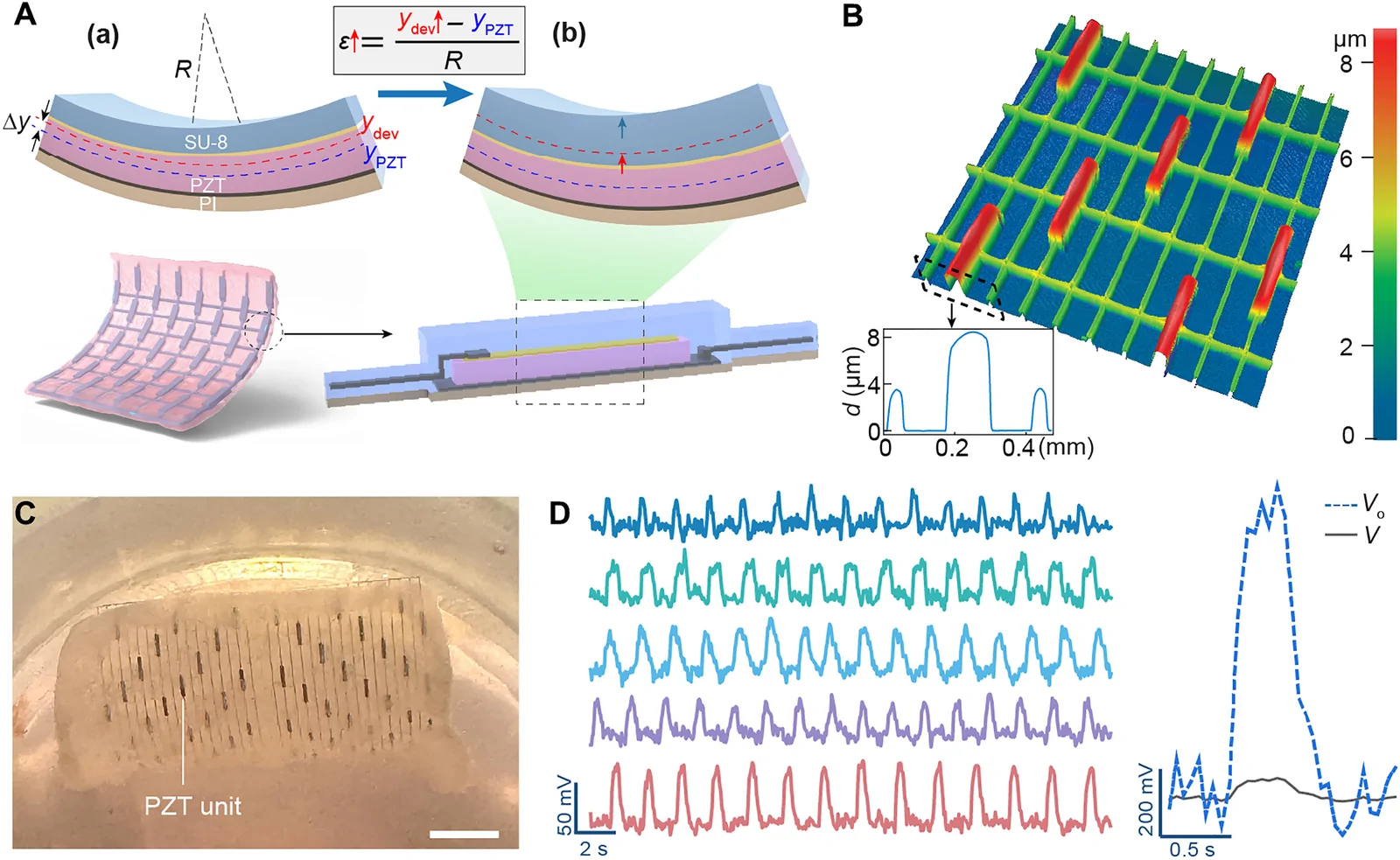
Local structural engineering in the mesh. (**A**) Schematics of locally thickening the device regions in the mesh architecture. (a) Illustration of the distance (∆*y*) between the mid-plane of the PZT layer (*y*_PZT_) and the neutral plane of the multilayer device structure (*y*_dev_). (b) Illustration showing that *y*_dev_ is shifted further away (red arrow) from *y*_PZT_ by increasing the thickness of the top SU-8 layer (blue arrow). (**B**) Surface topology of a fabricated mesh system showing thicker geometry at the device regions, (bottom) with the measured local thickness ∼8.5 μm. (**C**) A representative optical image showing the conformal wrapping of the mesh-tissue integration. Scale bar, 1 mm. (**D**) Representative measured voltage signals. Note that these recordings are not synchronized, due to the limited single-channel input in the measurement system. (Right) The calibrated actual signal amplitude $V_{o}$ (blue) relative to the measured value *V*, after accounting for parasitic capacitance effects introduced by the measurement system. Details for calibration can be found in note S4.

While the above analyses validated the consistency of the piezoelectric response and device functionality, they also pointed to a possible pathway for improving the output. Specifically, according to ε=∆y/R, the strain induced in the PZT layer depends on both the bending curvature *R* and the relative position of the PZT mid-plane with respect to the neutral plane of the multilayer structure. Given that the bending radius in a cardiac microtissue is governed by its intrinsic contractile dynamics, an effective strategy is to design the device so that the mid-plane of the PZT layer is positioned farther from the structural neutral plane of the multilayer device (Fig. 5Ab). This strategy for enhancement was evidently demonstrated by the bending tests (Fig. 3), where the relatively thick flexible substrate (e.g., ∼125 μm) effectively separated the PZT layer from the structural neutral plane (e.g., ∆y ∼ 47 μm). As a result, the induced strain, and hence the electric output, was substantially higher.

This motivated us to explore whether a similar strategy could be employed to enhance the electrical output of the mesh system. However, careful consideration is required. Specifically, while increasing the thickness of the mesh can effectively shift the PZT layer farther from the structural neutral plane, it also introduces a trade-off by increasing the bending stiffness of the system. Because bending stiffness scales strongly with thickness (e.g., with the cube of thickness), and because a microtissue sheet, unlike a whole organ tissue, can generate only limited bending force, such a trade-off is expected to hinder cardiac contraction. Moreover, this approach would deviate from the central theme of engineering a distributed harvesting system capable of seamless tissue integration, as increased stiffness has been shown to weaken tissue-device interface, promoting detachment and delamination.

We therefore designed a strategy to locally thicken the structure at the device regions while maintaining the original thickness of the interconnecting mesh ribbons (Fig. 5A). Because the device regions constitute only a small, repeating fraction of the overall architecture, this modification has minimal impact on the system-level mechanical properties. Such a strategy was previously employed in skin-wearable designs (*49*). In this way, the mesh can largely retain tissue-like mechanical properties, preserving an intimate tissue–device interface and minimizing perturbation to cardiac function. At the same time, global bending and tethering from the compliant ribbons are expected to facilitate deformation of the locally stiffened device region, potentially inducing sufficient local curvature such that the net effect remains constructive for enhanced strain generation.

To this end, we selectively increased the local thickness of the PZT units by lithographically defining an additional SU-8 encapsulation layer (∼5 μm) on top of them (Fig. 5B). The PZT layer thickness was also increased from 350 nm to 900 nm, which can contribute not only to the shift of the PZT mid-plane but also to an enhanced voltage output under a given strain (Eq. 2). As discussed above, this localized modification can largely preserve the overall mechanical compliance of the mesh system. Consequently, the modified mesh could still be readily embedded within a developing microtissue without impeding cardiac contractile dynamics (Fig. 5C, movie S1). The bending deformation during contraction in the microtissue was not reduced compared with the microtissue integrated with mesh without localized thickening (table S1), further indicating that the modification does not adversely affect tissue contraction. The added local SU-8 layer effectively shifted the PZT layer farther away from the structural neutral plane, increasing the offset distance ∆y from 0.12 to 0.75 μm. This increased offset is expected to enhance strain induction in PZT devices considering that the contractile dynamics in the microtissue were unimpeded. Consistent with this expectation, representative measurements showed an increased output voltage range of ∼30–50 mV (Fig. 5D). After accounting for parasitic capacitance in the measurement system (note S4), the calibrated actual signal range was ∼450–750 mV (Fig. 5D, right), representing over one order-of-magnitude improvement over the initial mesh design. This calibration was further validated by additional experiments (fig. S10). The estimated energy output per contraction cycle was ∼7 pJ (note S5), falling within the picojoule range characteristic of microscale biological processes. Given that the harvesting device has a volume comparable to that of a single cell, these results suggest compatibility with cellular-scale activity and potential utility in driving low-amplitude bio-emulated systems (*50*–*52*).

When considering the effective energy density based on the entire system volume, the mesh harvester achieves a value >10 μW/cm^3^ (note S5), more than an order of magnitude higher than typical values (e.g., sub-μW/cm^3^) reported in existing approaches based on the centralized paradigm (table S2). Note that the energy conversion efficiency in each PZT unit was estimated to be ∼3.5%, falling within the typical reported range for PZT-based harvesters (note S6). Therefore, the enhanced effective energy density does not result from an improvement in the intrinsic piezoelectric conversion efficiency, but rather from the engineered distributed mesh architecture, which minimizes the non-energy-converting volume while preserving the intrinsic energy conversion capability of the piezoelectric material. This result highlights the advantages of the distributed design, which substantially reduces the overall integration volume while maintaining efficient energy conversion.

We further analyzed how this local engineering contributed to the observed enhancement in signal amplitude. The average strain experienced in the PZT devices was estimated to be 7.7 × 10^−5^ based on Eq. 2, which relates electrical output to induced strain. This value represents ∼3 times of increase compared to the strain expected without local structural engineering in the mesh (∼2.0 × 10^−5^), as estimated from optical imaging of the global bending curvature of the microtissue (table S1). These results confirm that the local engineering was indeed helpful in improving strain transduction for enhancing electrical output. However, if the locally engineered PZT device fully conforms to the global bending curvature, it would predict a higher induced strain of ∼1.2 × 10^−4^ (table S1), corresponding to a calibrated output ∼940 mV. The discrepancy suggested that the local bending curvature of the PZT units deviated from the global curvature, consistent with our prior analysis and expectations. In other words, this local structural engineering, while showing effect on improving output, could not fully realize its theoretical potential. This limitation likely arises from the limited ability of the thin cardiac sheet to establish strong mechanical coupling, as evidenced by optical imaging (fig. S11).

The above limitation in the in vitro setting, nevertheless, points to the potential in vivo, where the thicker 3D tissue can provide substantially stronger mechanical coupling and bending. This is evident from many demonstrations that in vivo organ tissues can drive harvesting patches that rely on much thicker, nonporous substrates (*15*–*20*). By leveraging the mesh’s ability to embed and integrate seamlessly with tissue, multilayer and deep-tissue in vivo integration may become feasible, which can not only fully harness mechanical conversion but also realize 3D distributed harvesting networks that have yet to be demonstrated. In practice, potential approaches to realize in vivo integration include transplanting engineered tissues pre-embedded with mesh harvesters (*45*) or performing direct surgical implantation.

## DISCUSSION

We have explored the concept and potential of establishing a distributed harvesting network embedded in in vitro cardiac tissue environments. Unlike traditional implantable devices, this approach enables an intertwined, cell-attached integration that minimizes perturbation to the surrounding tissue. The long-term vision is that such networks could enable broad, 3D energy harvesting throughout tissue, thereby expanding the effective energy source volume and improving system stability without compromising tissue function. This strategy may ultimately advance the concept of bio-hybridization or “cyborg” for bioelectronic integration.

Our study provides a pilot exploration of this concept by examining system design and function in an in vitro cardiac tissue system, yielding several notable findings. First, we demonstrate seamless integration of the harvester network with microtissues by leveraging a mesh electronic scaffold engineered with tissue-like properties. The system realizes tissue-scale integration with cell-level intimacy, providing compelling evidence for the feasibility of distributed energy harvesting and transmission across tissue. Second, we validate the feasibility of directly coupling individual energy harvesting units to cells within the tissue. While the energy output is currently limited by the thin tissue construct that constrains effective mechanical transduction, it nonetheless achieves an effective energy density more than an order of magnitude higher than that of in vivo harvesters based on the centralized paradigm. These results point to significant potential for translation to in vivo systems, where stronger coupling and multilayer 3D integration could further enhance performance. Third, the framework developed here, which includes a tissue-like interconnecting network and distributed cell-interfacing harvesting units, can be extended to other biohybrid energy harvesting systems in which energy modalities are not restricted to mechanical sources. Ultimately, such systems could enable the retrieval or conversion of cellular energy, potentially emulating the broadly distributed ATP-based processes in biological systems, to achieve greater energy sustainability.

## MATERIALS AND METHODS

### Fabrication of PZT units

A precursor solution was prepared by mixing 0.992 g zirconium(IV) propoxide and 0.545 g titanium(IV) isopropoxide with 1.03 g acetic acid and 1.6 g isopropyl alcohol, followed by the addition of 1.686 g lead(II) acetate trihydrate. The mixture was heated to 90°C to ensure complete dissolution of the salt and then cooled down to room temperature. Subsequently, 1.5 g acetic acid and 0.56 g deionized (DI) water were added and stirred for 5 min. Finally, 2.1 g isopropyl alcohol and 1.05 g acetic acid were added and the solution was stirred for 24 hours to complete the sol formation.

A silicon substrate (capped with 100 nm SiO_2_) was coated with Pt/Ti (300 nm/20 nm) by magnetron sputtering (AJA ORION 8) and used for PZT deposition using the sol–gel method. The prepared solution was spin-coated at 3000 rpm for 60 s. Pyrolysis and pre-crystallization were carried out at 180°C for 20 min and 400°C for 20 min, respectively, using a rapid thermal annealing furnace (Solaris 100) in air. After 3 consecutive depositions (total thickness ∼350 nm), the sample was annealed at 700°C for 10 min. The PZT/Pt device patterns were defined sequentially by inductively coupled plasma reactive ion etching (ICP-RIE; Oxford PlasmaPro 300 RIE), using AZ 4625 photoresist as the sacrificial masks.

### Mesh fabrication

AZ 4625 photoresist was defined on PZT/Pt units as the protective layer for releasing the PZT/Pt units from the substrate. The sample was immersed in hydrofluoric acid (HF) for partial removal of the SiO_2_ underneath and rinsed by DI water. The photoresist was removed by acetone. A thermal tape was used to peel off the PZT/Pt units.

100-nm Ge was deposited by electron-beam evaporation on another substrate as the releasing layer. Poly(pyromellitic dianhydride-co-4,4′-oxydianiline) amic acid (precursor PI) solution was spin-coated on the Ge layer at 6000 rpm for 60 s and baked at 90°C for 2 min. The peel-off PZT/Pt units were transferred onto this precursor PI layer, followed by heating the sample to 90°C to release the PZT/Pt units from the thermal tape. The substrate was then heated at 150°C, 200°C, and 250°C (each for 30 min) to fully cure the PI layer. Standard photolithography, metal deposition, and lift-off processes were used to define the top electrodes (50/10 nm Au/Cr) of the PZT/Pt units. A layer of SU-8 2002 photoresist with openings was defined by photolithography to provide “via” access to the top and bottom electrodes of the Au/PZT/Pt units, followed by standard lithographic processes to define interconnects (100/10 nm Pt/Ti). Another layer of SU-8 2002 was lithographically defined to encapsulate the contacts and interconnects. The mesh structure was finally defined by ICP-RIE etching (Oxford PlasmaPro 300 RIE) using photoresist (AZ 4625) as the sacrificial mask. The device used for performance evaluation was fabricated following the same process. The top electrodes were omitted and instead realized as part of top interconnects through enlarged vias.

### Cell culture and tissue integration

The cardiomyocytes were differentiated from commercially available human embryonic stem cells (hESCs, WAe009-A, H9, WiCell, NIH registration no. 0062, female). Differentiation was initiated (day 0) by replacing the culture medium with RPMI 1640 (Gibco) supplemented with 1% B27 minus insulin (Gibco) and 8 μM CHIR99021 (Tocris Bioscience). After 24 hours (day 1), the medium was replaced with fresh RPMI 1640 containing 1% B27 minus insulin. On day 3, the medium was changed to RPMI 1640 with 1% B27 minus insulin supplemented with 5 μM IWR-1-endo (Cayman Chemical). Subsequently, the medium was replaced with RPMI 1640 containing 1% B27 minus insulin on day 5 and with RPMI 1640 containing 1% B27 (Gibco) on day 7. Thereafter, the cells were maintained in RPMI 1640 supplemented with 1% B27, with medium changes every other day.

The mesh system was released from substrate by dissolving the Ge sacrificial layer in 1% H_2_O_2_ solution (30 min) before integrating with cardiomyocytes. The released mesh was thoroughly rinsed by DI water and sterilized by immersion in 70% ethanol for 1 hour. Then the mesh system was rinsed by Dulbecco’s phosphate-buffered saline (DPBS) solution, followed by the overnight treatment of Poly-D-lysine (0.1 mg/ml, Gibco) in an incubator. Matrigel (50 μl) was coated on the mesh after aspirating Poly-D-lysine solution. Cardiomyocytes at day 10 to 12 after differentiation were resuspended in RPMI 1640 plus 1% B27 with 10 μM Y-27632 ROCK inhibitor and seeded onto the mesh and incubated for 24 hours. Then the cells were maintained using RPMI 1640 plus 1% B27 with daily change of medium.

### Electrical measurements

The PZT units were electrically poled at 150 kV/cm at 150°C. The electrical outputs were measured using Keithley 6514 A. For electromechanical testing, bending was applied using a mechanical testing stage (ESM303; Mark-10 Inc).

### Material characterizations

The cross section of sol-gel PZT film was imaged by a scanning electron microscope (SEM, JSM-7001F; JEOL). The crystallization was characterized by x-ray diffraction (Panalytical XRD). The surface topology of mesh system was characterized by 3D optical profiler (Zygo Nexview). The local thickness was measured by a stylus profiler (Dektak, Bruker).

### Fluorescence staining and imaging

Rhodamine 6G (Sigma-Aldrich) was mixed into the SU-8 solution for the encapsulation layer to obtain the mesh electronics for fluorescence imaging. The cardiac microtissues with mesh electronics were first fixed with 4% PFA/PBS (v/v) solution and then permeabilized with 0.2% Triton-X/PBS (w/v) solution (Thermo Fisher Scientific). The fixed samples were washed with DPBS, followed by blocking in 10% normal goat serum solution (Thermo Fisher Scientific). 4′,6-diamidino-2-phenylindole (DAPI, diluted in the blocking solution with a ratio of 1:5000, Invitrogen), and Phalloidin-Alexa Fluor 488 (diluted in the blocking solution with a ratio of 1:400, Invitrogen) were used for co-staining for 3 hours at room temperature. The samples were washed with DPBS three times before imaging. Imaging was performed using confocal microscopy (Nikon), acquiring z-stack images of the wavy laminar tissues.

Device biocompatibility was evaluated using a LIVE/DEAD assay kit (Invitrogen). Calcein AM and ethidium homodimer-1 (EthD-1) were diluted in DPBS to final concentrations of 1 and 2.5 μM, respectively. Samples were incubated with the staining solution at 37°C for 30 min and rinsed once with DPBS before imaging.

## Supplementary Materials

### This PDF file includes:

Supplementary Text Tables S1 and S2 Figs. S1 to S11 Legend for movie S1 References
