# Supplementary material for: A biohybrid mesh harvester for distributed energy harvesting in living tissues
Source: Sci Adv. 2026 Oct 7;12(41):eaei5963. doi: 10.1126/sciadv.aei5963 (PMC13644788; doi:10.1126/sciadv.aei5963)
Supplement: Supplementary file 1 [file sciadv.aei5963_sm.pdf]

Science Advances
AAAS

Supplementary Materials for

A biohybrid mesh harvester for distributed energy harvesting in living tissues

Siqi Wang *et al.*

Corresponding author: Jun Yao, juny@umass.edu

Sci. Adv. **12**, eaei5963 (2026)
DOI: 10.1126/sciadv.aei5963

The PDF file includes:

Supplementary Text
Tables S1 and S2
Figs. S1 to S11
Legend for movie S1
References

Other Supplementary Material for this manuscript includes the following:

Movie S1

Supplementary Text

Supplementary Note 1

Piezoelectric electrical analysis

The constitutive equation of piezoelectric material gives the relationship (*40*) between the stress σ_{ij}, strain ε_{ij}, electric field E_i, and electrical displacement D_i

$$\begin{bmatrix}\sigma_{11}\\ \sigma_{22}\\ \sigma_{33}\\ \sigma_{23}\\ \sigma_{31}\\ \sigma_{12}\end{bmatrix}=\begin{bmatrix}c_{11} & c_{12} & c_{13} & 0 & 0 & 0\\ c_{12} & c_{11} & c_{13} & 0 & 0 & 0\\ c_{13} & c_{13} & c_{33} & 0 & 0 & 0\\ 0 & 0 & 0 & c_{44} & 0 & 0\\ 0 & 0 & 0 & 0 & c_{44} & 0\\ 0 & 0 & 0 & 0 & 0 & (c_{11}-c_{12})/2\end{bmatrix}\begin{bmatrix}\varepsilon_{11}\\ \varepsilon_{22}\\ \varepsilon_{33}\\ 2\varepsilon_{23}\\ 2\varepsilon_{31}\\ 2\varepsilon_{12}\end{bmatrix}-\begin{bmatrix}c_{11} & c_{12} & c_{13} & 0 & 0 & 0\\ c_{12} & c_{11} & c_{13} & 0 & 0 & 0\\ c_{13} & c_{13} & c_{33} & 0 & 0 & 0\\ 0 & 0 & 0 & c_{44} & 0 & 0\\ 0 & 0 & 0 & 0 & c_{44} & 0\\ 0 & 0 & 0 & 0 & 0 & (c_{11}-c_{12})/2\end{bmatrix}\begin{bmatrix}E_1\\ E_2\\ E_3\end{bmatrix} \quad \text{(Eq. S1)}$$

$$\begin{bmatrix}D_1\\ D_2\\ D_3\end{bmatrix}=\begin{bmatrix}0 & 0 & 0 & 0 & e_{15} & 0\\ 0 & 0 & 0 & e_{15} & 0 & 0\\ e_{31} & e_{31} & e_{33} & 0 & 0 & 0\end{bmatrix}\begin{bmatrix}\varepsilon_{11}\\ \varepsilon_{22}\\ \varepsilon_{33}\\ 2\varepsilon_{23}\\ 2\varepsilon_{31}\\ 2\varepsilon_{12}\end{bmatrix}+\begin{bmatrix}k_{11} & 0 & 0\\ 0 & k_{22} & 0\\ 0 & 0 & k_{33}\end{bmatrix}\begin{bmatrix}E_1\\ E_2\\ E_3\end{bmatrix} \quad \text{(Eq. S2)}$$

where c_{ij}, e_{ij}, and k_{ij} are the stiffness tensor, piezoelectric parameter, and the dielectric constant of the PZT, respectively. Based on the strain of the PZT layer along *y* direction $\varepsilon_{22}=0$, together with $\sigma_{33}=0$ from the traction-free top surface of the structure, the electric displacement can be further derived as $D_3=\bar{e}\varepsilon_{11}+\frac{\bar{k}V}{Nd}$, where $\bar{e}=e_{31}-(c_{13}/c_{33})e_{33}$ is the effective piezoelectric constant, $\bar{k}=k_{33}+e_{33}^2/c_{33}$ the effective piezoelectric constant, *V* the total voltage across *N* PZT units in series, and d the thickness of PZT layer.

The current is expressed as $I=-A\dot{D}_3$, where *A* denotes the area of the PZT unit. For the measurement instrument with internal resistance *R*, the voltage follows $I=V/R=-A\dot{D}_3$, i.e.

$$\frac{dV}{dt}=\frac{N\cdot\bar{e}\cdot d}{A\cdot R\cdot\bar{k}}V=-\frac{N\bar{e}d}{\bar{k}}\frac{d\varepsilon_{11}}{dt} \quad \text{(Eq. S3)}$$

For the boundary condition $V_{t=0}=0$, the voltage is given by (*17*)

$$V=\frac{-\bar{e}Nd}{\bar{k}}e^{\frac{-Nd}{AR\bar{k}}t}\int_0^t\frac{d\varepsilon_{11}}{dt}e^{\frac{Nd}{AR\bar{k}}t}dt \quad \text{(Eq. S4)}$$

which corresponds to the voltage-strain relationship shown in Eq.1 of the main text.

The current output is given by $I=A\dot{D}_3$, where $D_3=\bar{e}\varepsilon_{11}+\frac{\bar{k}V}{Nd}$. Therefore

$$I=A\dot{D}_3=A\bar{e}\dot{\varepsilon}_{11}+\frac{\bar{k}\dot{V}}{Nd} \quad \text{(Eq. S5)}$$

The second term is negligible at peak current, yielding the linear relationship between the rate of strain change and the current as described in Eq.2 of the main text.

Supplementary Note 2

Mechanical analysis of PZT units on the flexible substrate

One end of the substrate was fixed, and the compression force was applied to the other end along the longitudinal axis using a mechanical stage to induce buckling. Based on previous studies (*53*), the buckling profile can be described as by the out-of-plane displacement *w*:

$$w(x) = \frac{A}{2}(1 + cos(\frac{2\pi x}{L})), -\frac{L}{2} < x < \frac{L}{2} \quad \text{(Eq. S6)}$$

where *x* denotes the coordinate along the length *L* of the structure defined with origin at the center, and *A* is the buckling amplitude (i.e. apex).

The in-plane displacement ΔL accommodated by buckling is obtained by:

$$\Delta L = \frac{1}{2}\int_{-L/2}^{L/2}(w'(x))^2 dx = \frac{A^2\pi^2}{4L} \quad \text{(Eq. S7)}$$

Minimization of total energy with respect to *A* yields (*42*):

$$A = \frac{2}{\pi}\sqrt{L\Delta L - \frac{\pi^2 t_{PI}^2}{3}} \quad \text{(Eq. S8)}$$

where t_{PI} is the thickness of the commercial PI film. For the PI film under buckling, ${t_{PI}}^2 \ll L\Delta L$ giving the approximate scaling:

$$A \approx \frac{2}{\pi}\sqrt{L\Delta L} \quad \text{(Eq. S9)}$$

For an N-layer multilayer device structure subject to pure bending, the neutral mechanical plane y_{dev}, defined as the distance from the bottom surface of the multilayer device structure:

$$y_{dev} = \frac{\sum_{i=1}^{N}\left(\sum_{j=1}^{i} t_j - t_i/2\right)}{\sum_{i=1}^{N}\bar{E}_i t_i} \quad \text{(Eq. S10)}$$

where $\bar{E}_i$ and t_i are the plane-strain modulus and thickness of the *i*th layer, respectively. The effective bending stiffness of the multilayer device structure is

$$\overline{EI}_{dev} = \sum_{i=1}^{N}\bar{E}_i t_i\left[{t_i}^2/3 + \left(\sum_{j=1}^{i} t_i - y_{dev}\right)\left(\sum_{j=1}^{i} t_j - y_{dev} - t_i\right)\right] \quad \text{(Eq. S11)}$$

The curvature w'' of a commercial PI film dominated buckling structure can be evaluated at *x*=0 as $w'' = -4\pi\sqrt{\Delta L/L}/\text{L}$. To incorporate the mechanical contribution of the multilayer device structure, a correction factor $\overline{EI}_{PI}/\overline{EI}_{dev}$ is introduced to rescale the local curvature, under the assumption that the bending moment is preserved, i.e., $\overline{EI}_{PI}w'' = \overline{EI}_{dev}{w_{dev}}''$. The axial membrane strain at the center of PZT is then given by

$$\varepsilon_{11} = (\overline{EI}_{PI}/\overline{EI}_{dev})w''\Delta y \quad \text{(Eq. S12)}$$

where $\overline{EI}_{PI} = \bar{E}_{PI}t_{PI}^3/12$ and Δy is the distance from the mid-plane of PZT layer to the mechanical neutral plane of the multilayer device structure (i.e., $y_{PZT} - y_{dev}$).

Supplementary Note 3

Mechanical analysis on biohybrid mesh

As shown in Figure S5a, the relation between the bending radius R and the cylindrical projection of the biohybrid mesh onto the substrate X can be described as

$$X = \begin{cases} R\sin(X_0/R) & , R \geq 2X_0/\pi \\ R & , R < 2X_0/\pi \end{cases} \qquad \text{(Eq. S13)}$$

where X_0 is the length of the unbending mesh. The bending radius R and corresponding strain ε in PZT can be further related as:

$$\varepsilon = \Delta y\,/R = (y_{PZT} - y_{dev})/R \qquad \text{(Eq. S14)}$$

where Δy represents the distance between the mid-plane of the PZT layer (y_{PZT}) and the neural plane of the entire multilayer device structure (y_{dev}). The strain change $\Delta\varepsilon = \varepsilon_2 - \varepsilon_1 = \Delta y\,/R_2 - \Delta y\,/R_1$ in the PZT device between phase 1 (relaxation) and phase 2 (contraction) determines the voltage generated from the biohybrid mesh.

Table S1. **Estimated stain in mesh with or without local structural engineering through optical tracking.** Cases 1 and 2 correspond to mesh-tissue integrations described in Fig. 4 and Fig. 5 of the main text, respectively.

**Case1**	Without local engineering	Δy (nm)	X_1 (μm)	R_1 (μm)	X_2 (μm)	R_2 (μm)	$\boldsymbol{\varepsilon_1}$	$\boldsymbol{\varepsilon_2}$	$\boldsymbol{\Delta\varepsilon}$
		125	8170	8620	6200	6000	1.5×10^{-5}	2.1×10^{-5}	6.4×10^{-6}
**Case2**	With local engineering	Δy (nm)	X_1 (μm)	R_1 (μm)	X_2 (μm)	R_2 (μm)	$\boldsymbol{\varepsilon_1}$	$\boldsymbol{\varepsilon_2}$	$\boldsymbol{\Delta\varepsilon}$
		750	2060	2060	1550	1550	3.6×10^{-4}	4.8×10^{-4}	1.2×10^{-4}
	(If) without local engineering	Δy (nm)	X_1 (μm)	R_1 (μm)	X_2 (μm)	R_2 (μm)	$\boldsymbol{\varepsilon_1}$	$\boldsymbol{\varepsilon_2}$	$\boldsymbol{\Delta\varepsilon}$
		125	2060	2060	1550	1550	6.0×10^{-5}	8.0×10^{-5}	2×10^{-5}

Supplementary Note 4

Calibrating the electrical output

The parasitic capacitance (C_p) arising from the measurement system redistributes the induced polarization charges, resulting in a discrepancy between the measured (V) and actual electrical output (V_o) from the mesh system when integrated with tissues. Specifically, the measured voltage is reduced to $V = V_o \frac{C/M}{C/M + C_p}$, where M (= 4) is the number of PZT units connected in series and C the capacitance of a single PZT unit. The parasitic capacitance C_p was estimated to be ~350 pF based on the analysis presented in Fig. 3 of the main text.

For the biohybrid mesh system demonstrated in Fig. 5, C was measured to be ~100 pF (fig. S4), and the calculated calibration factor $\frac{C/M + C_p}{C/M}$ is ~15, consistent with values validated by additional experiments (fig. S9), yielding a calibrated output V_o of 450~750 mV for measured output V of 30~50 mV after accounting for the parasitic effect.

The electrical output in Fig. 4 was calibrated using the same approach with C measured ~256 pF because of the difference in PZT film thickness, resulting in a calibrated factor $\frac{C/M + C_p}{C/M}$ of ~6.5.

Supplementary Note 5

Power density of the biohybrid mesh harvester

The electrical energy output per contraction cycle is estimated by $E = \frac{C \cdot V_o^2}{2}$, where C is the device capacitance and V_o is the open-circuit voltage. For the device consisting of four PZT units connected in series (each with measured capacitance ~100 pF), the equivalent capacitance is 25 pF. The open-circuit voltage corresponds to the calibrated output voltage with an optimal value ~750 mV. These values lead to an estimated E ~7 pJ per cycle.

The effective power density is $P = \frac{E \cdot f}{V}$, where V is the total volume of the device (e.g., including interconnecting ribbons) and f the contractile frequency (~0.4 Hz). V is estimated ~2.2×10^{-7} cm^3. These values lead to an estimated power density ~13 μW/cm^3.

Table S2. **Effective energy density of piezoelectric energy harvesters accounting for packaging volume.**

Ref.	Material	Integration method	Encapsulation		Device volume⁋	Power density	Interface	Interfacing method
			Top	Bottom				
(54)	PMN-PT	Nonporous flexible substrate	40 µm PDMS+ 5 µm parylene-C	100 µm PET+ 5 µm parylene-C	> 1.5 cm^3	< 0.6 µW/cm^3 *	Porcine heart	Surgical suturing
(55)	PVDF-TrFE	Nonporous flexible substrate	20 µm PDMS	20 µm PDMS	0.130 cm^3	0.28 µW/cm^3†	Porcine heart	Endovascular delivery
(56)	KNN	Nonporous flexible substrate	Not specified	125 µm PET	> 0.15 cm^3	< 0.58 µW/cm^3 ‡	Porcine heart	Surgical suturing
(57)	PVDF-TrFE	Nonporous flexible substrate	700 µm PDMS+ 150 µm Kapton	700 µm PDMS+ 150 µm Kapton	3.98 cm^3	0.25 µW/cm^3 *	Dog heart	Surgical suturing
(17)	PZT	Nonporous flexible substrate	1.2 µm PI	70 µm PI film	0.038 cm^3	1.3 µW/cm^3 *	Bovine heart	Surgical suturing
(15)	ZnO nanowire	Nonporous flexible substrate	Insulating wax	50 µm Kapton film	> 0.003 cm^3	< 45 pW/cm^3*	Rat heart	Surgical suturing
(58)	PVDF-TrFE	Nonporous flexible substrate	50 µm PI film	50 µm PI film	0.280 cm^3	107 nW/cm^3 *	Porcine aorta	Surgical suturing
(59)	PVDF	Nonporous flexible substrate	50 µm PI film	50 µm PI film	0.165 cm^3	0.24 µW/cm^3 *	Porcine aorta	Surgical suturing
(60)	ZnO nanowire	Nonporous flexible substrate	Flexible polymer	50 µm Kapton film	> 0.003 cm^3	< 45 pW/cm^3‡	Hamster skin	Physical attachment
(61)	PVDF-TrFE	Thin film substrate	300 nm parylene-C	1.25 µm PI film	0.0004 cm^3	2.8 µW/cm^3	Human skin	Epidermal attachment
(62)	ZnO nanowire	Nonporous flexible substrate	Not specified	Eyelash glue	0.0006 cm^3	< 2.5 nW/cm^3‡	Human skin	Epidermal attachment
(63)	MoSe2	Nonporous flexible substrate	PDMS	PET+PDMS	0.242 cm^3	< 0.286 nW/cm^3†	Human skin	Epidermal attachment
(64)	PZT composite	Stretchable substrate	0.25 mm PDMS	0.68 mm PDMS	1.43 cm^3	1.3 µW/cm^3 ‡	Human skin	Epidermal attachment
(65)	PVDF	Yarn-based fabrics	Device without packaging		0.94 cm^3	< 0.002 µW/cm^3 ‡	Human skin	Epidermal attachment
(66)	DL alanine	Stretchable substrate	~5 µm PDMS	~5 µm PDMS	0.01 cm^3	< 0.4 nW/cm^3 ‡	Swine muscle	Surgical implantation
(67)	PVDF	Stretchable substrate	2 µm parylene-C	2 µm parylene-C	0.0063 cm^3	0.15 µW/cm^3 ‡	Mouse leg	Surgical implantation
(31)	PVDF	Nonporous cantilevers	~50 µm PDMS	~50 µm PDMS	~ 0.02 cm^3	~1.38 µW/cm^3*	In vitro cardiac microtissue	Surface tissue attachment
(68)	PVDF	Flexible nanofiber mat	Direct cell culture	~5 µm PDMS	~ 0.0004 cm^3	~0.46 µW/cm^3‡	In vitro cardiac cell sheet	Surface cell sheet growth
(69)	PMN–PT	Nonporous thin film	Direct cell culture	PDMS for fixture	~ 0.0002 cm^3	~5.7 nW/cm^3*	In vitro cardiac cell sheet	Planar cell sheet growth
This work	PZT	Porous mesh architecture	2 µm SU-8	1.5 µm PI	2.2×10^{-7} cm^3	13 µW/cm^3	In vitro cardiac microtissue	Tissue embedment

Note: The background suggests different interfaces.
⁋: The device volume reported in this table refers to the total volume involved in energy harvesting, including the active piezoelectric material, encapsulation layers, and other essential structural components.
* The value was estimated using device volume and power/energy output.
† The value was estimated based on in vitro testing data, with the measured output closely matching that observed in vivo.
‡ The value was estimated using device volume, current and voltage output.

Supplementary Note 6

Energy conversion efficiency in individual PZT unit

Since piezoelectric energy harvesters convert mechanical energy into electrical energy, we estimated the mechanical energy involved in each bending cycle. The total bending strain energy stored in an *N*-layer device unit can be expressed as $U = \frac{1}{2} wL \sum_{i=1}^{N} \bar{E}_i {\varepsilon_i}^2 t_i$ (*61*), where w and L are the width and length of the PZT unit, respectively; $\bar{E}_i$, t_i are the plane-strain modulus and thickness of the ith layer, respectively. The strain in the PZT layer ($\varepsilon_{PZT} \sim 7.7 \times 10^{-5}$) was estimated from measured electrical output, while the strains in the remaining layers were determined based on their relative distances from the neutral mechanical plane.

The energy conversion efficiency, η, was then calculated as the ratio of the electrical energy generated to the total bending strain energy stored in the multilayer device unit during one contraction cycle: $\eta = \frac{E}{U}$, where E is the electrical energy generated per contraction cycle by a single PZT unit, as determined experimentally (~1.75 pJ) with details shown in Supplementary Note 5. Using this analysis, η was estimated to be ~3.5 %, which falls within the typical reported range (0.7-7%) for PZT-based harvesters (*17, 70, 71*).

Supplementary Figures

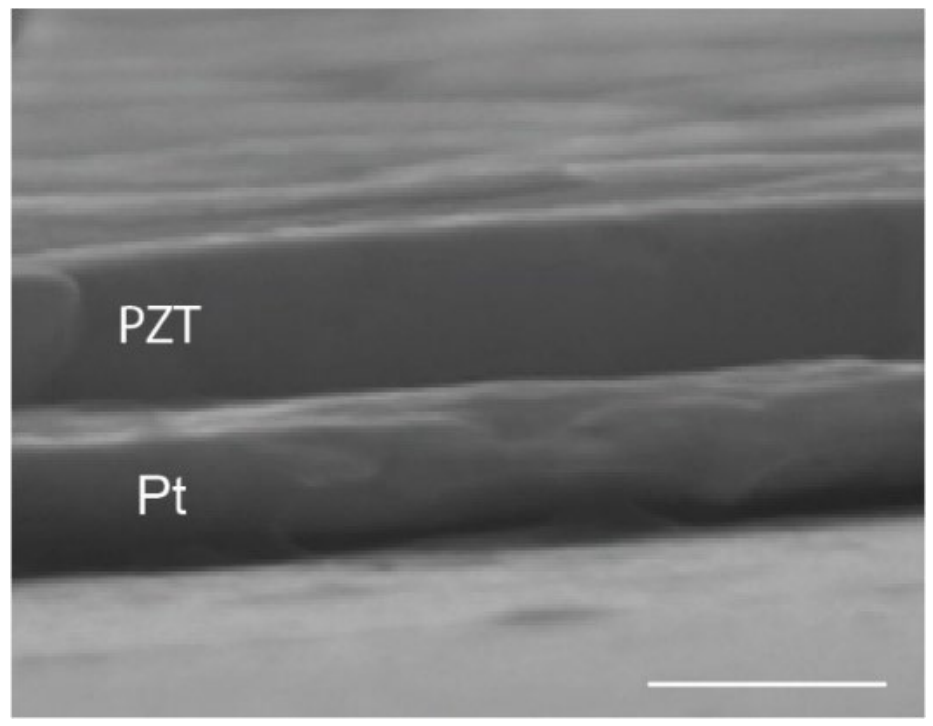

Fig. S1. Cross-section scanning electron microscope (SEM) image of a prepared sol-gel PZT film deposited on a Pt substrate. Scale bar, 500 nm.

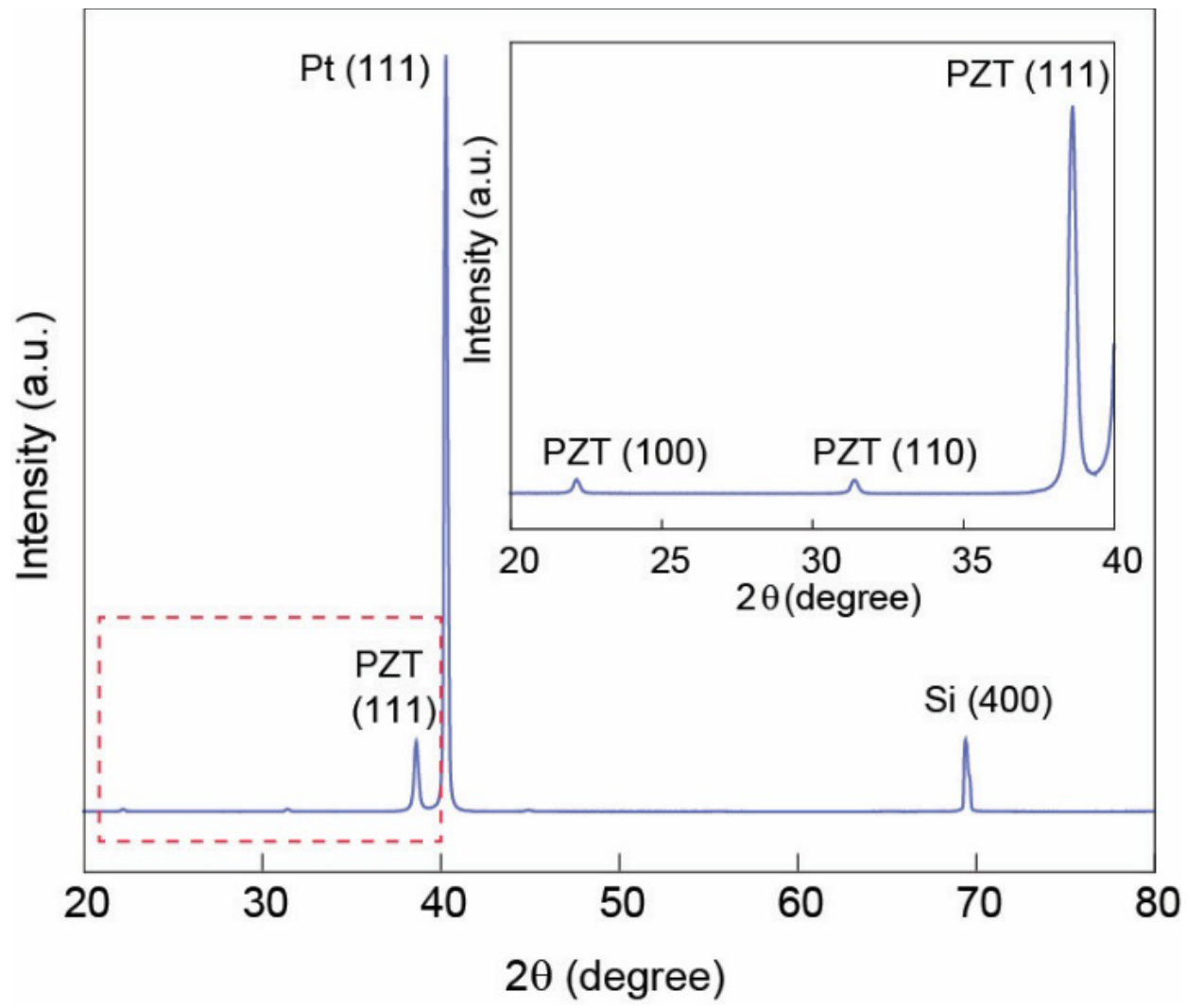

Fig. S2. **X-ray diffraction (XRD) spectrum of the sol-gel PZT film deposited on Pt substrate.** The inset shows the zoom-in feature in the dashed box. The degree of preferred orientation of the polycrystal PZT film can be evaluated using the Lotgering factor $F = \frac{P-P_0}{1-P_0}$ (*72*), where P is the ratio of the summed intensities of the preferred (111) reflections to the total diffraction intensity of the measured PZT film, and P_0 is the corresponding ratio for randomly oriented PZT powder (powder diffraction file card #01-070-4264) as the random reference. The calculated $F_{(111)}$ is 92.6%, demonstrating a dominant (111) orientation, consistent with prior report (*35*), which has been reported to enhance piezoelectric properties (*36*).

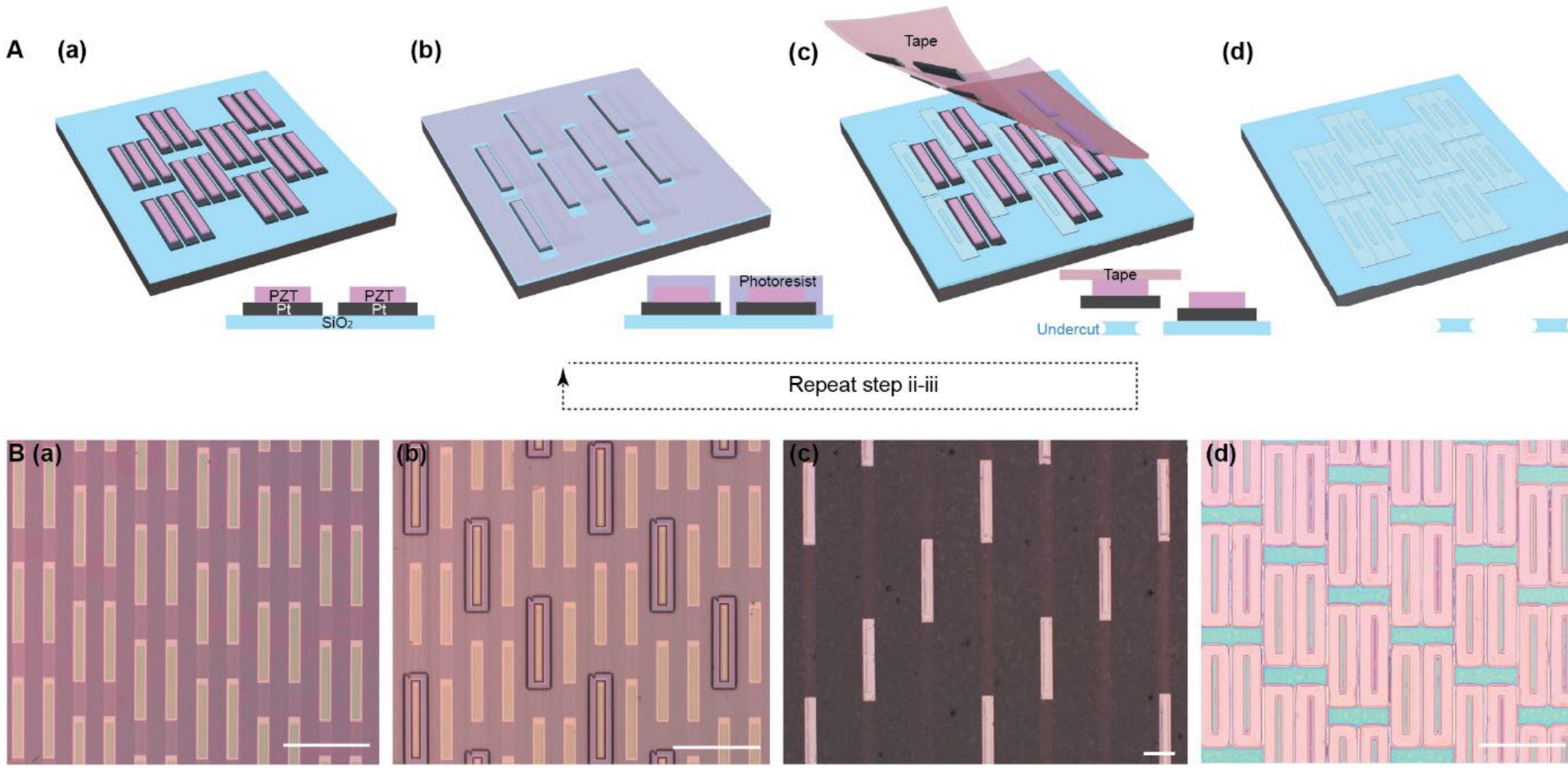

Fig. S3. **(A)** Schematics of the selective peel-and-transfer processes. (a) Multiple arrays of PZT/Pt units are defined on SiO_2/Si wafer by dry etching method. (b) Selectively exposing the surrounding SiO_2 substrate of one array of PZT/Pt units by photolithography using photoresist as the protective layer. (c) After immersion in HF to induce SiO_2 undercut beneath the selected array of PZT/Pt units, the array is peeled off and transferred with a thermal release tape. (d) Repeating steps ii-iii leads to the consecutively peeling off all the PZT/Pt arrays. **(B)** Optical images of (a) as-prepared multiple arrays of PZT/Pt units, (b) selective exposure of an array of PZT/Pt units with photoresist, (c) the selectively peeled-off array of PZT/Pt units on a thermal release tape, and (d) the blank substrate with all arrays of PZT/Pt units consecutively peeled off and transferred. Scale bars, 500 μm.

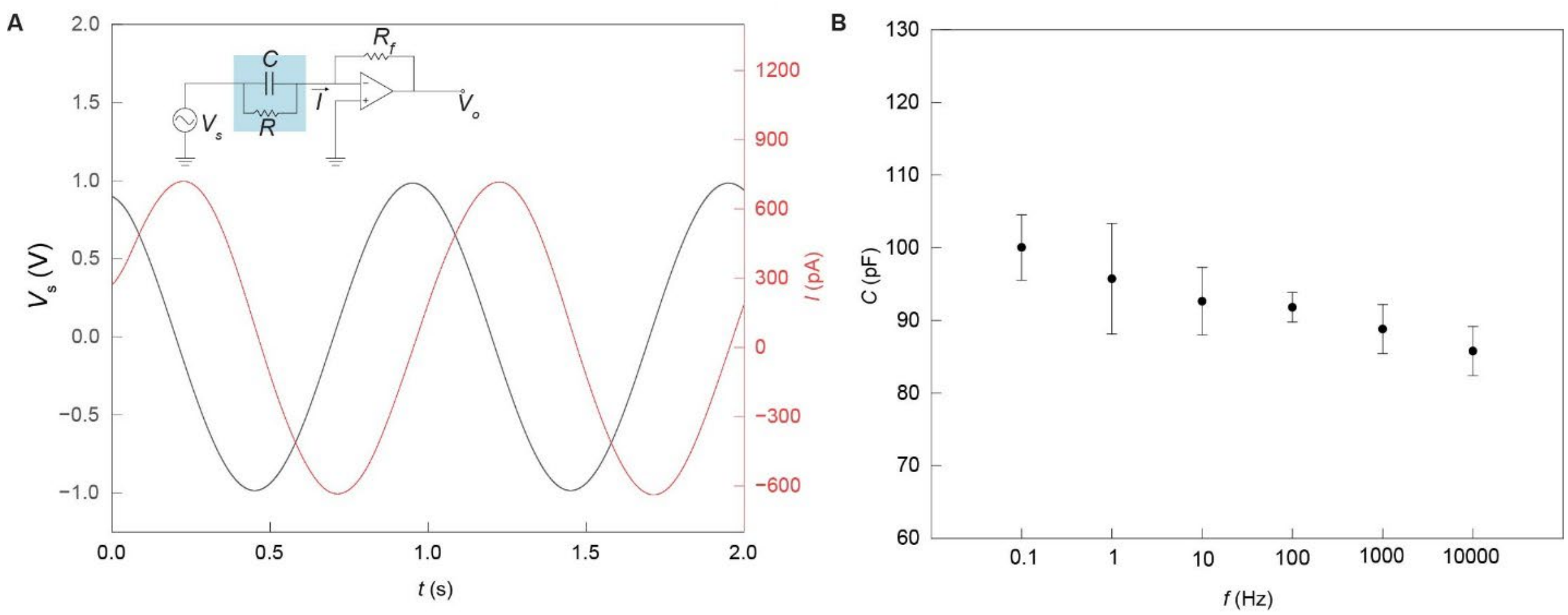

Fig. S4. Measurement of capacitance in the PZT unit. (Inset schematic) A sinusoidal AC voltage V_s is applied across the PZT unit, which is represented by an equivalent RC circuit. The resulting AC current response *I*, together with its phase relative to the applied voltage, is recorded. *I* can be obtained through an amplifier circuit as V_o/R_f. The capacitance can be extracted by $C = \frac{I \cdot sin\theta}{2\pi f V_s} = \frac{V_o \cdot sin\theta}{2\pi f V_s R_f}$, where θ is the phase lead of the current relative to the voltage, *f* is the frequency of exciting voltage; *I*, V_o, and V_s here represent the amplitudes of the AC signals. **(A)** Representative input V_s at 1 Hz and the corresponding current response *I* from a PZT unit. (**B**) The extracted capacitance *C* at different input frequency from 0.1 to 10000 Hz. The extracted capacitance is ~100 pF near the cardiac contraction frequency. Data is presented as mean ± SD (n = 5).

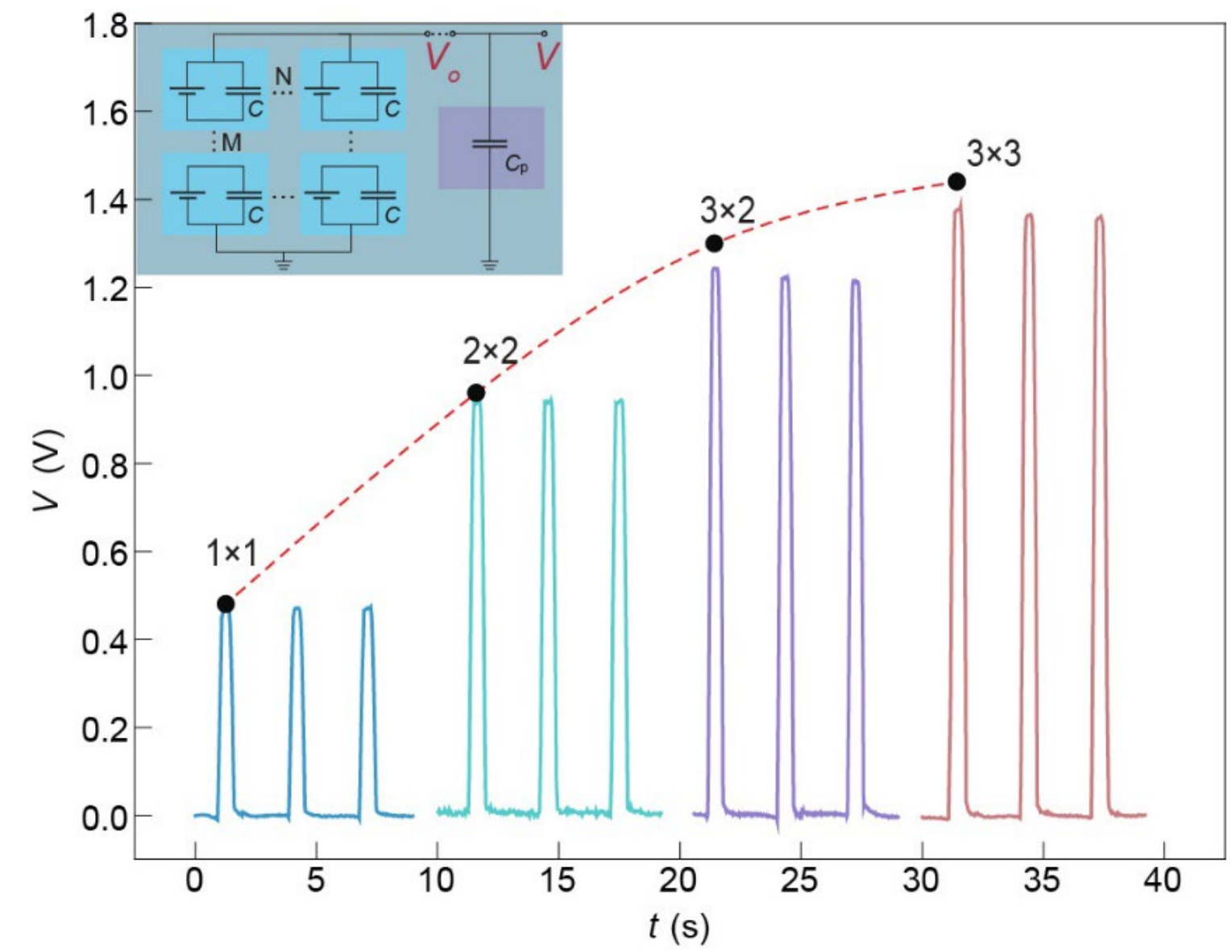

Fig. S5. Measured voltage V from multiple PZT units connected with hybrid series-parallel configurations, i.e., M PZT units are connected in series to form a group and N such groups are further connected in parallel. The inset schematic shows the equivalent circuit, accounting for the parasitic capacitance C_p introduced by the measurement system. Each PZT unit is represented by a voltage source connected with its intrinsic capacitance C. V_o is the actual voltage output from the N×M array. The red dashed curve illustrates the measured output V predicted by the model from $V = V_o \frac{\frac{N}{M} \cdot C}{\frac{N}{M} \cdot C + C_p}$, where the capacitance of a single PZT unit C was measured to be ~ 100 pF and the parasitic capacitance C_p was previously obtained to be ~ 350 pF (Fig. 3F). The predicted V (dashed line) shows a good consistency with the actual measurement data (color curves).

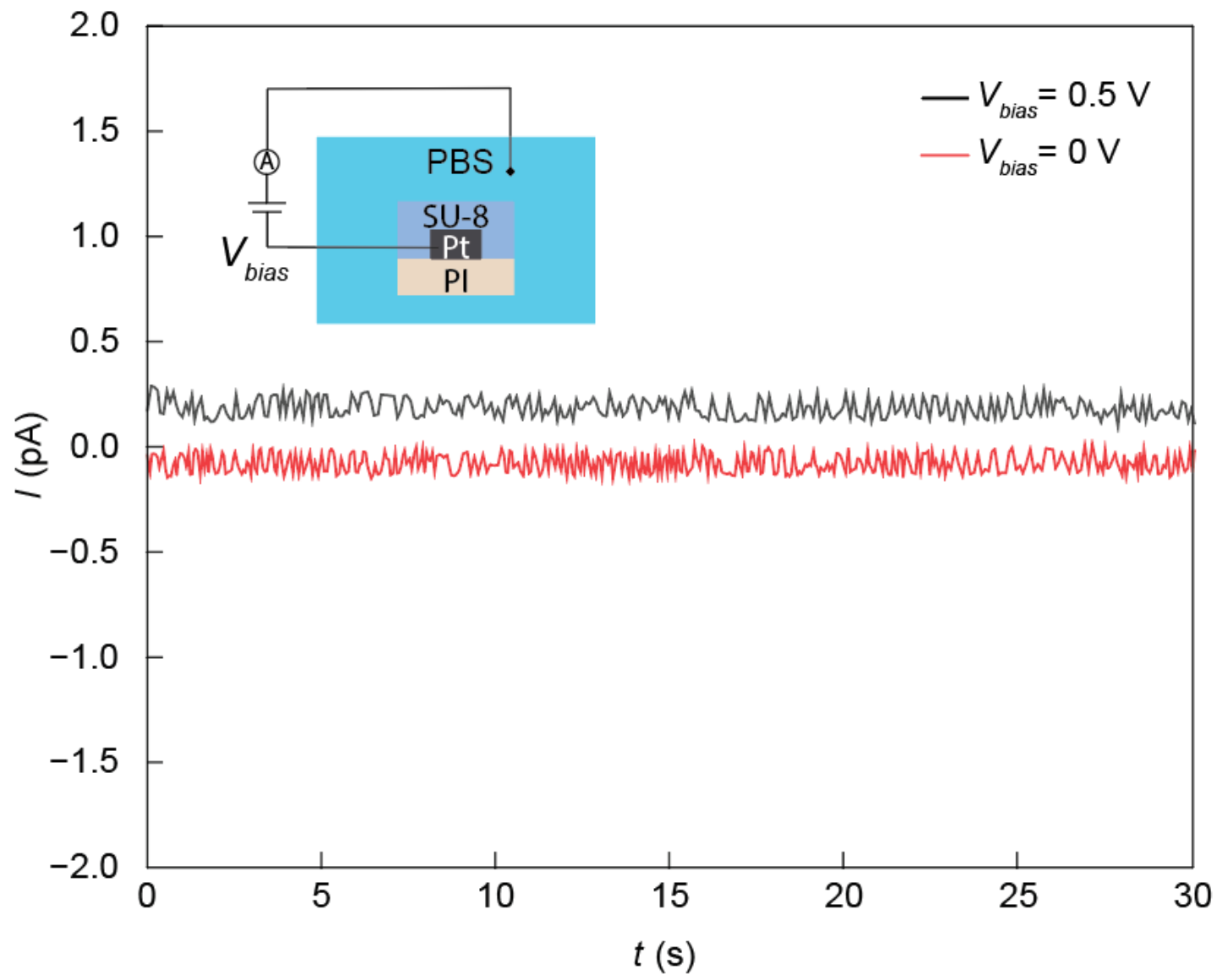

Fig. S6. Leakage-current measurement of a mesh device after 64 days of immersion in cell culture media. A bias voltage (V_{bias}) was applied between a Pt reference electrode immersed in phosphate-buffered saline (PBS) and the encapsulated interconnect. The measurement revealed that applying a V_{bias} of 0.5 V resulted in a leakage current I ~ 0.25 pA, which is at the noise floor of the measurement system and therefore considered negligible. These results indicate that the encapsulation provides effective electrical insulation, minimizing potential contributions from ionic or electrochemical activity within the cardiac microtissue

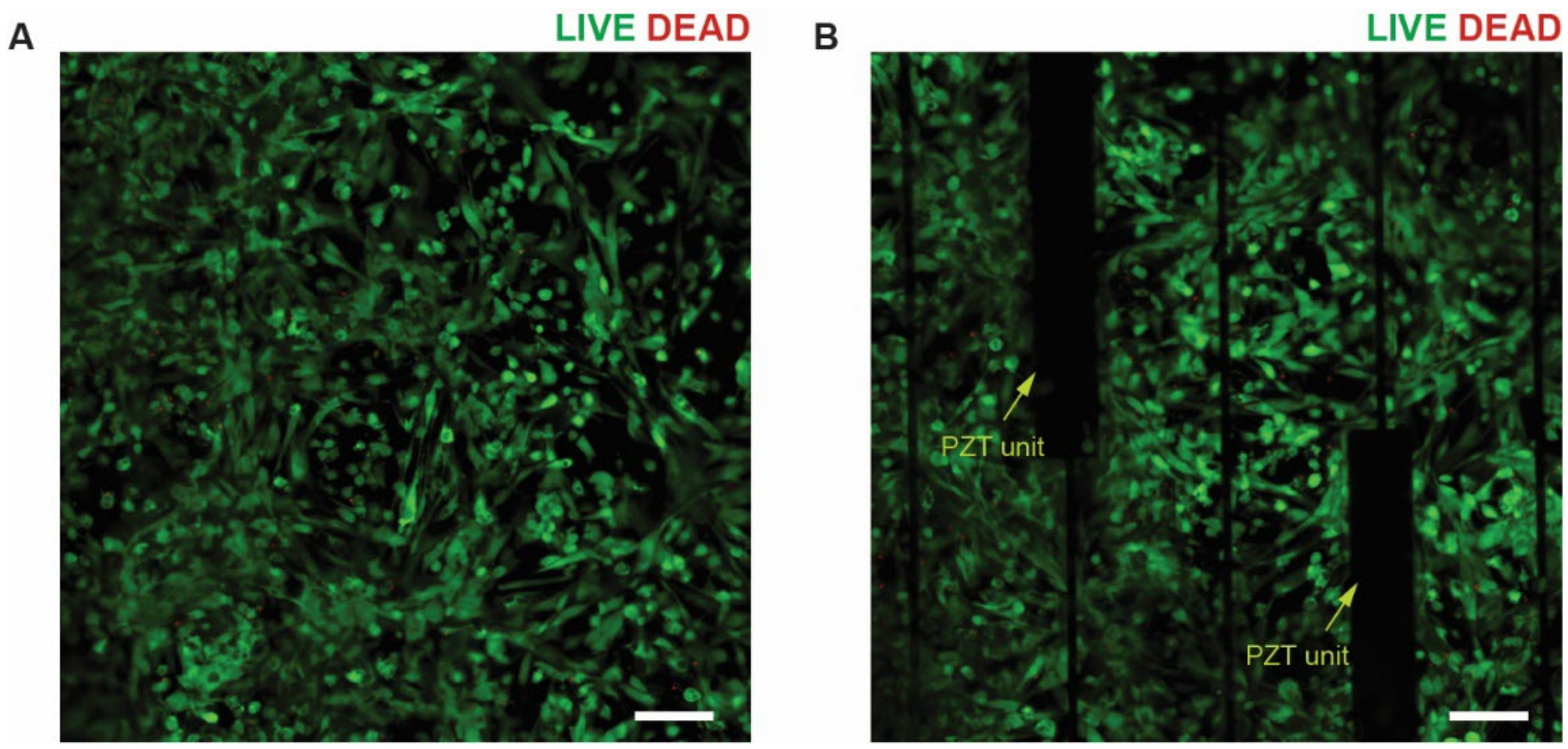

Fig. S7. Representative LIVE/DEAD fluorescence images of cardiac microtissues 3 days after seeding **(A)** on bare substrate and **(B)** on a mesh harvester containing PZT units. Live cells were stained with Calcein-AM (green), while dead cells were stained with EthD-1 (red). The images show predominantly live cells and no obvious accumulation of dead cells near the PZT regions. Note that the blocked cell image at the PZT and interconnect regions does not suggest absence of cell attachment, since the images were acquired from the bottom side of a transparent substrate. Scale bars, 100 μm.

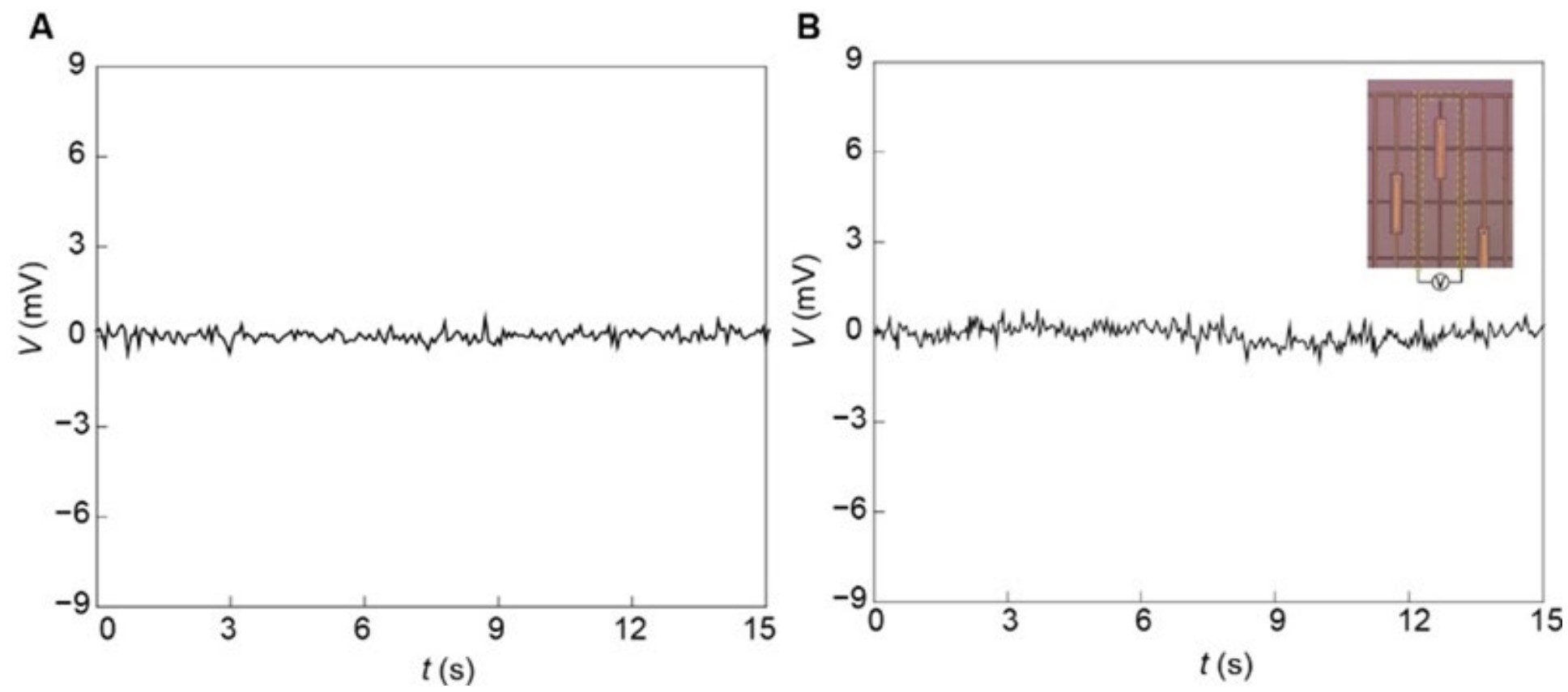

Fig. S8. **(A)** Voltage recording from tissue-integrated PZT mesh harvesters before the spontaneous contraction occurs. No electrical signal peak was observed, suggesting that the recorded signals (Fig. 4E, main text) did not originate from endogenous electrical activity. **(B)** Electrical recordings from a mesh with only metal interconnects connected (without PZT units connected, inset) and mechanically deformed in an aqueous environment. No electrical signal peak was observed, suggesting that the recorded signals did not originate from mechanical artifacts such as deformation of the interconnects or measurement setup itself.

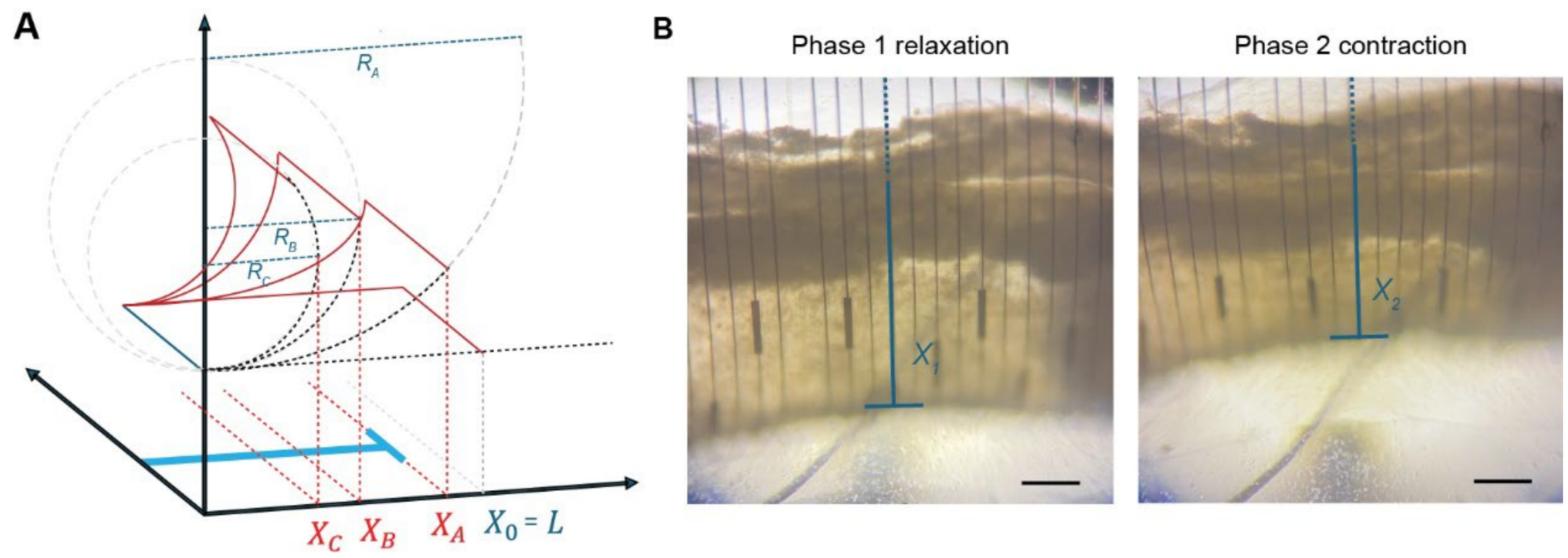

Fig. S9. **(A)** Schematic of the model to measure the overall bending radius R of the biohybrid mesh via optical tracking of its projections X. The bending radii (R_A, R_b, R_C) under different various bending states are illustrated. For $R \geq 2X_0/\pi$, $X = R\sin(X_0/R)$; for $R < 2X_0/\pi$, $X = R$, where X_0 is the length of the unbending mesh. **(B)** Representative images of the biohybrid mesh at different tissue contractile phases. Phase 1 and 2 correspond to relaxation and contraction, respectively. Blue lines indicate the cylindrical projections of the mesh at phase 1 and 2, from which the bending radius R_1 and R_2 can be estimated according to relationship in (A). Scale bars, 500 μm. Numerical values corresponding to different mesh-tissue integrations are listed in Table S1 under Supplementary Note 3.

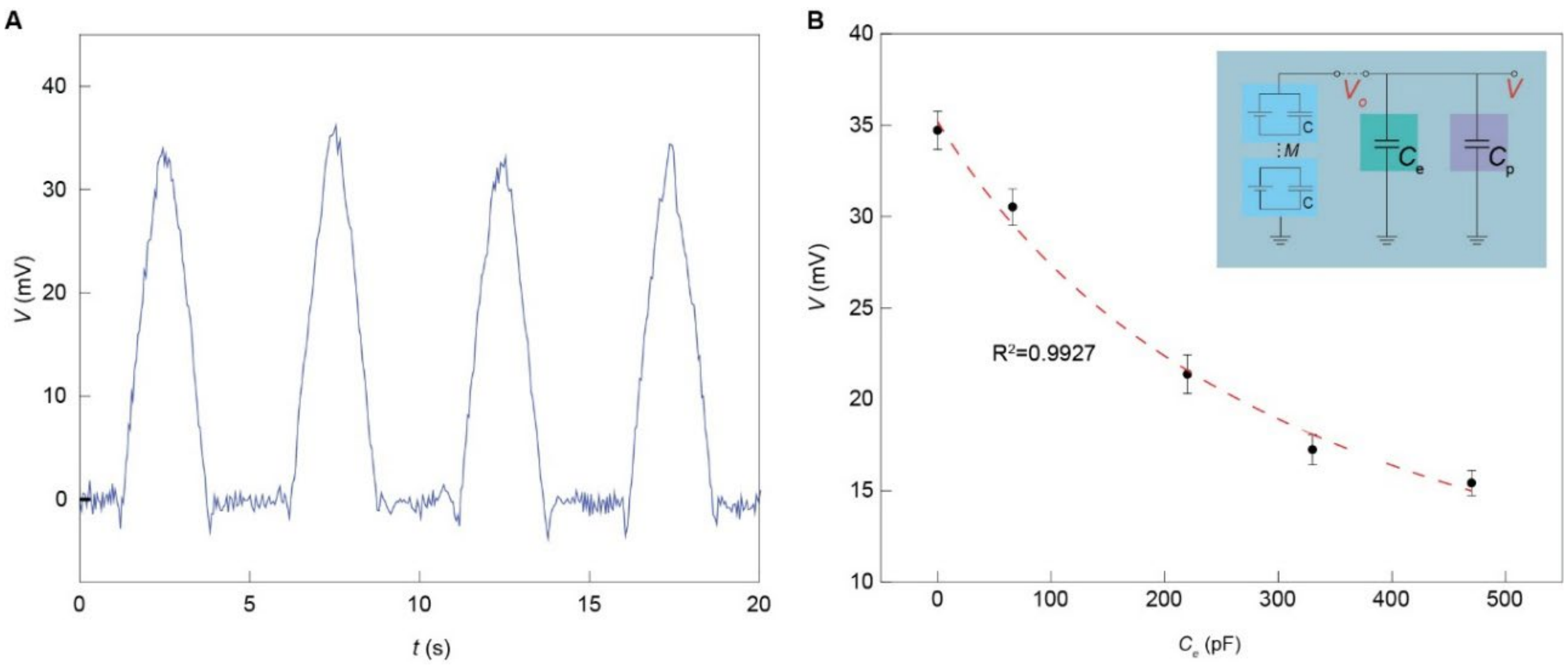

Fig. S10. **(A)** For cross-validating the calibration of the signals measured in tissue integration, we fabricated a PZT device with the same structural and electrical configuration as those integrated with the tissue. By applying mechanical strain (e.g., bending the PI substrate), we generated a measured output voltage of ~ 35 mV, comparable to the voltage levels obtained during tissue integration. **(B)** We connected the PZT device (containing *M* = 4 units) in parallel with an external capacitor while varying its capacitance. The output voltage was then measured under the same applied strain for different external capacitance values. Based on the equivalent circuit (inset), the measured voltage *V* is related to the actual voltage V_o by $V = V_o \frac{C/M}{C/M+C_p+C_e}$, where *C/M*, C_p, and C_e denote the capacitance of the PZT device, the parasitic capacitance, and the external capacitance, respectively. The measured data are well described by this relationship. From the fit (dashed line), we obtained a calibration factor of $\frac{C/M+C_p}{C/M}$ = 14.0$\pm$1.0, consistent with the calculated calibration factor of ~15 used for the tissue-integration experiments (Supplementary Note 4), where the measured output of 30–50 mV was calibrated to an actual output of 450–750 mV.

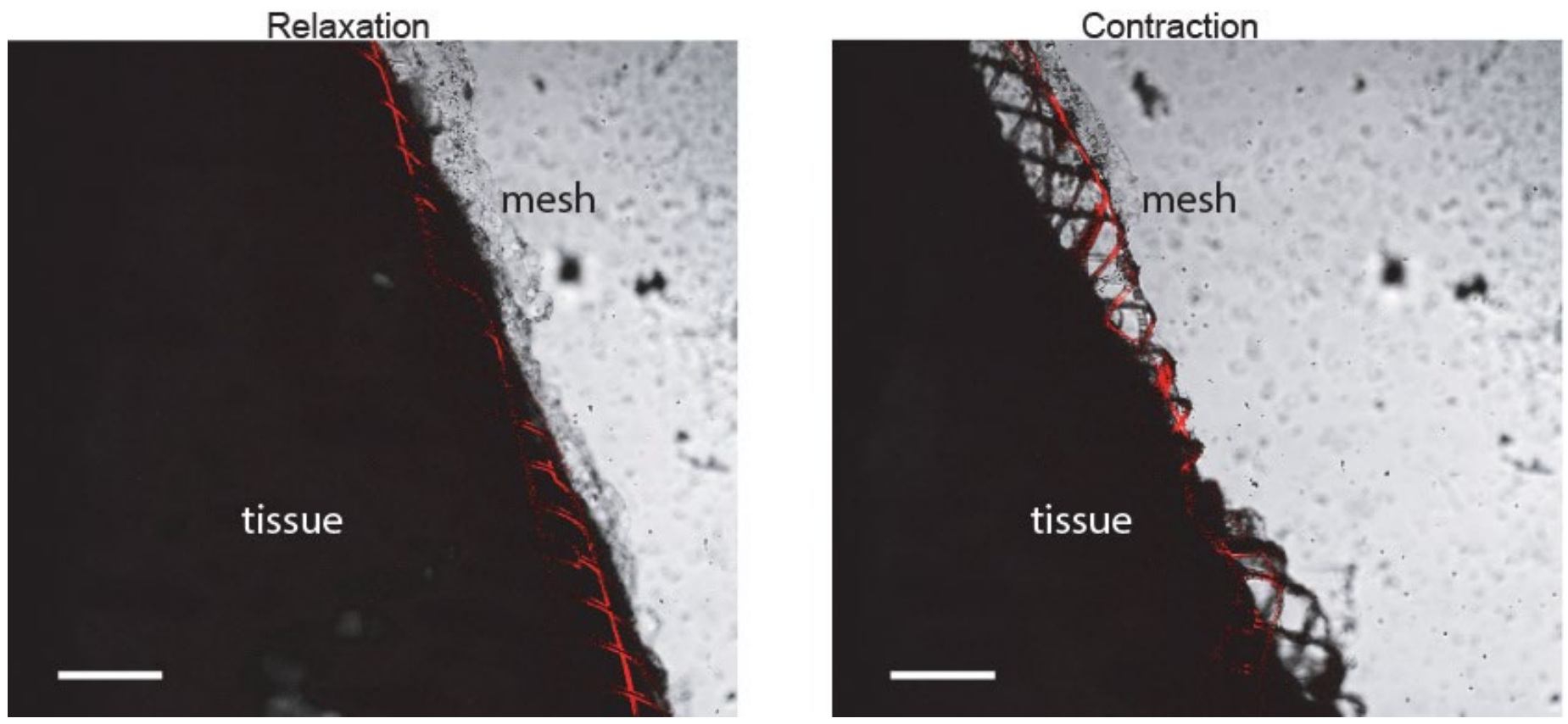

Fig. S11. Fluorescence microscopy images showing mesh-tissue integration across different contractile phases. The mesh (red) was applied with structural engineering of local thickening. Note that this sample is a different one from that in Fig. 5 of the main text. This example reveals that occasional local detachment of the mesh from the cardiac microtissue was observed during contraction, indicating that the shallow embedment (due to the thin tissue sheet) can limit effective mechanical coupling. Scale bars, 500 μm.

Movie S1. Contractile dynamics of a cardiac microtissue embedded with the mesh harvester.

REFERENCES

1. Y. Song, J. Min, W. Gao, Wearable and implantable electronics: moving toward precision therapy. *ACS Nano* **13**, 12280–12286 (2019).

2. C. Yang, B. Wang, J. Wan, A. Kamath, F. You, B. Tian, Quantifying the global eco-footprint of wearable healthcare electronics. *Nature* **649**, 73–82 (2026).

3. T. R. Ray, J. Choi, A. J. Bandodkar, S. Krishnan, P. Gutruf, L. Tian, R. Ghaffari, J. A. Rogers, Bio-integrated wearable systems: A comprehensive review. *Chem. Rev.* **119**, 5461–5533 (2019).

4. Y. Yang, W. Gao, Wearable and flexible electronics for continuous molecular monitoring. *Chem. Soc. Rev.* **48**, 1465–1491 (2019).

5. A. Chortos, J. Liu, Z. A. Bao, Pursuing prosthetic electronic skin. *Nat. Mater.* **15**, 937–950 (2016).

6. X. Tang, H. Shen, S. Zhao, N. Li, J. Liu, Flexible brain-computer interfaces. *Nat. Electron.* **6**, 109–118 (2023).

7. T. Zhou, H. Yuk, F. Hu, J. Wu, F. Tian, H. Roh, Z. Shen, G. Gu, J. Xu, B. Lu, X. Zhao, 3D printable high-performance conducting polymer hydrogel for all-hydrogel bioelectronic interfaces. *Nat. Mater.* **22**, 895–902 (2023).

8. V. S. Mallela, V. Ilankumaran, N. S. Rao, Trends in cardiac pacemaker batteries. *Indian Pacing Electrophysiol. J.* **4**, 201–212 (2004).

9. D. C. Bock, A. C. Marschilok, K. J. Takeuchi, E. S. Takeuchi, Batteries used to power implantable biomedical devices. *Electrochim. Acta* **84**, 155–164 (2012).

10. L. Wang, Y. Zhang, P. G. Bruce, Batteries for wearables. *Natl. Sci. Rev.* **10**, nwac062 (2023).

11. Y. Song, D. Mukasa, H. Zhang, W. Gao, Self-powered wearable biosensors. *Acc. Mater. Res.* **2**, 184–197 (2021).

12. X. Liu, T. Ueki, H. Gao, T. L. Woodard, K. P. Nevin, T. Fu, S. Fu, L. Sun, D. R. Lovley, J. Yao, Microbial biofilms for electricity generation from water evaporation and power to wearables. *Nat. Commun.* **13**, 4369 (2022).

13. S. Wang, L. Lin, Z. L. Wang, Nanoscale triboelectric-effect-enabled energy conversion for sustainably powering portable electronics. *Nano Lett.* **12**, 6339–6346 (2012).

14. Q. Zheng, B. J. Shi, Z. Li, Z. L. Wang, Recent progress on piezoelectric and triboelectric energy harvesters in biomedical systems. *Adv. Sci.* **4**, 1700029 (2017).

15. Z. Li, G. Zhu, R. Yang, A. C. Wang, Z. L. Wang, Muscle-driven in vivo nanogenerator. *Adv. Mater.* **22**, 2534–2537 (2010).

16. Q. Zheng, B. Shi, F. Fan, X. Wang, L. Yan, W. Yuan, S. Wang, H. Liu, Z. Li, Z. L. Wang, In vivo powering of pacemaker by breathing-driven implanted triboelectric nanogenerator. *Adv. Mater.* **26**, 5851–5856 (2014).

17. C. Dagdeviren, B. D. Yang, Y. Su, P. L. Tran, P. Joe, E. Anderson, J. Xia, V. Doraiswamy, B. Dehdashti, X. Feng, B. W. Lu, R. Poston, Z. Khalpey, R. Ghaffari, Y. G. Huang, M. J. Slepian, J. A. Rogers, Conformal piezoelectric energy harvesting and storage from motions of the heart, lung, and diaphragm. *Proc. Natl. Acad. Sci. U.S.A.* **111**, 1927–1932 (2014).

18. D. H. Kim, H. J. Shin, H. Lee, C. K. Jeong, H. Park, G.-T. Hwang, H.-Y. Lee, D. J. Joe, J. H. Han, S. H. Lee, J. Kim, B. Joung, K. J. Lee, In vivo self-powered wireless transmission using biocompatible flexible energy harvesters. *Adv. Funct. Mater.* **27**, 1700341 (2017).

19. N. Li, Z. Yi, Y. Ma, F. Xie, Y. Huang, Y. Tian, X. Dong, Y. Liu, X. Shao, Y. Li, L. Jin, J. Liu, Z. Xu, B. Yang, H. Zhang, Direct powering a real cardiac pacemaker by natural energy of a heartbeat. *ACS Nano* **13**, 2822–2830 (2019).

20. P. Cheng, S. Dai, Y. Liu, Y. Li, H. Hayashi, R. Papani, Q. Su, N. Li, Y. Dai, W. Liu, H. Hu, Z. Liu, L. Jin, N. Hibino, Z. Wen, X. Sun, S. Wang, An intrinsically stretchable power-source system for bioelectronics. *Device* **2**, 100216 (2024).

21. Z. Liu, Y. Hu, X. Qu, Y. Liu, S. Cheng, Z. Zhang, Y. Shan, R. Luo, S. Weng, H. Li, H. Niu, M. Gu, Y. Yao, B. Shi, N. Wang, W. Hua, Z. Li, Z. L. Wang, A self-powered intracardiac pacemaker in swine model. *Nat. Commun.* **15**, 507 (2024).

22. L. Dong, A. B. Closson, C. Jin, Y. Nie, A. Cabe, D. Escobedo, S. Huang, I. Trase, Z. Xu, Z. Chen, M. D. Feldman, J. X. J. Zhang, Multifunctional pacemaker lead for cardiac energy harvesting and pressure sensing. *Adv. Healthc. Mater.* **9**, e2000053 (2020).

23. B. Tian, J. Liu, T. Dvir, L. Jin, J. H. Tsui, Q. Qing, Z. Suo, R. Langer, D. S. Kohane, C. M. Lieber, Macroporous nanowire nanoelectronic scaffolds for synthetic tissues. *Nat. Mater.* **11**, 986–994 (2012).

24. G. Hong, X. Yang, T. Zhou, C. M. Lieber, Mesh electronics: A new paradigm for tissue-like brain probes. *Curr. Opin. Neurobiol.* **50**, 33–41 (2018).

25. G. Hong, C. M. Lieber, Novel electrode technologies for neural recordings. *Nat. Rev. Neurosci.* **20**, 330–345 (2019).

26. X. Yang, T. Zhou, T. J. Zwang, G. Hong, Y. Zhao, R. D. Viveros, T. M. Fu, T. Gao, C. M. Lieber, Bioinspired neuron-like electronics. *Nat. Mater.* **18**, 510–517 (2019).

27. Q. Li, K. Nan, P. Le Floch, Z. Lin, H. Sheng, T. S. Blum, J. Liu, Cyborg organoids: Implantation of nanoelectronics via organogenesis for tissue-wide electrophysiology. *Nano Lett.* **19**, 5781–5789 (2019).

28. H. Gao, Z. Wang, F. Yang, X. Wang, S. Wang, Q. Zhang, X. Liu, Y. Sun, J. Kong, J. Yao, Graphene-integrated mesh electronics with converged multifunctionality for tracking multimodal excitation-contraction dynamics in cardiac microtissues. *Nat. Commun.* **15**, 2321 (2024).

29. T. Zhou, G. Hong, T. M. Fu, X. Yang, T. G. Schuhmann, R. D. Viveros, C. M. Lieber, Syringe-injectable mesh electronics integrate seamlessly with minimal chronic immune response in the brain. *Proc. Natl. Acad. Sci. U.S.A.* **114**, 5894–5899 (2017).

30. X. Yang, C. Forró, T. L. Li, Y. Miura, T. J. Zaluska, C. T. Tsai, S. Kanton, J. P. McQueen, X. Chen, V. Mollo, F. Santoro, S. P. Pasca, B. Cui, Kirigami electronics for long-term

electrophysiological recording of human neural organoids and assembloids. *Nat. Biotechnol.* **42**, 1836–1843 (2024).

31. B. Xu, X. Lin, W. Li, Z. Wang, W. Zhang, P. Shi, Cell generator: A self-sustaining biohybrid system based on energy harvesting from engineered cardiac microtissues. *Adv. Funct. Mater.* **27**, 1606169 (2017).

32. B. Rubehn, T. Stieglitz, In vitro evaluation of the long-term stability of polyimide as a material for neural implants. *Biomaterials* **31**, 3449–3458 (2010).

33. R. W. Schwartz, T. J. Boyle, S. J. Lockwood, M. B. Sinclair, D. Dimos, C. D. Buchheit, Sol-gel processing of PZT thin films: A review of the state-of-the-art and process optimization strategies. *Integr. Ferroelectr.* **7**, 259–277 (1995).

34. T. Hase, T. Sakuma, K. Amanuma, T. Mori, A. Ochi, Y. Miyasaka, Difference in microstructure between PZT thin films on Pt/Ti and those on Pt. *Integr. Ferroelectr.* **8**, 89–98 (1995).

35. M. Xiao, S. Li, Z. Lei, Study of (111)-oriented PZT thin films prepared by a modified sol–gel method. *J. Mater. Sci. Mater. Electron.* **26**, 4031–4037 (2015).

36. Z. Ren, S. Deng, J. Shao, Y. Si, C. Zhou, J. Luo, T. Wang, J. Li, J. Li, H. Liu, X. Qi, P. Wang, A. Yin, L. Wu, S. Yu, Y. Zhu, J. Chen, S. Das, J. Wei, Z. Chen, Ultrahigh-power-density flexible piezoelectric energy harvester based on freestanding ferroelectric oxide thin films. *Nat. Commun.* **16**, 3192 (2025).

37. H. Gao, B. Yin, S. Wu, X. Liu, T. Fu, C. Zhang, J. Lin, J. Yao, Deterministic assembly of three-dimensional suspended nanowire structures. *Nano Lett.* **19**, 5647–5652 (2019).

38. H. Gao, F. Yang, K. Sattari, X. Du, T. D. Fu, S. Fu, X. Liu, J. Lin, Y. Sun, J. Yao, Bioinspired two-in-one nanotransistor sensor for the simultaneous measurements of electrical and mechanical cellular responses. *Sci. Adv.* **8**, eabn2485 (2022).

39. H. Takahashi, M. Nakayama, T. Shimizu, M. Yamato, T. Okano, Anisotropic cell sheets for constructing three-dimensional tissue with well-organized cell orientation. *Biomaterials* **32**, 8830–8838 (2011).

40. A. Marino, G. G. Genchi, E. Sinibaldi, G. Ciofani, Piezoelectric effects of materials on bio-interfaces. *ACS Appl. Mater. Interfaces* **9**, 17663–17680 (2017).

41. J. Sirohi, I. Chopra, Fundamental understanding of piezoelectric strain sensors. *J. Intell. Mater. Syst. Struct.* **11**, 246–257 (2000).

42. J. Song, Y. Huang, J. Xiao, S. Wang, K. C. Hwang, H. C. Ko, D. H. Kim, M. P. Stoykovich, J. A. Rogers, Mechanics of noncoplanar mesh design for stretchable electronic circuits. *J. Appl. Phys.* **105**, 123516 (2009).

43. C. K. Lee, T. C. O'Sullivan, Piezoelectric strain rate gages. *J. Acoust. Soc. Am.* **90**, 945–953 (1991).

44. Y. Cui, H. Yu, Z. Abbas, Z. Wang, L. Wang, D. Wang, PZT composite film preparation and charaterization using a method of sol-gel and electrohydrohynamic jet printing. *Micromachines* **14**, 918 (2023).

45. J. Aoyama, R. Liu, X. H. Zhang, A. Y. Zhu, P. Luanpaisanon, N. Velayutham, J. C. Garbern, F. Cao, I. Barrera, H. Fandl, M. Sokol, S. Dasariraju, E. S. Gil, E. Aleksi, T. Amanuma, J. J. Saucerman, F. Chen, J. Liu, R. T. Lee, Flexible nanoelectronics reveal arrhythmogenesis in transplanted human cardiomyocytes. *Science* **390**, eadw4612 (2025).

46. S. B. Park, C. T. Sun, Effect of electric field on fracture of piezoelectric ceramics. *Int. J. Fract.* **70**, 203–216 (1993).

47. K. Yamashita, M. Okuyama, Characterization of piezoelectric property of sol-gel PZT thin films and its improvement by poling. *IEEJ Trans. Sens. Micromachines* **124**, 124–128 (2004).

48. H. Miyabuchi, T. Yoshimura, S. Murakami, N. Fujimura, Characterization of direct piezoelectric effect in 31 and 33 modes for application to vibration energy harvester. *Jpn. J. Appl. Phys.* **50**, 09ND17 (2011).

49. W. Wang, S. Wang, R. Rastak, Y. Ochiai, S. Niu, Y. Jiang, P. K. Arunachala, Y. Zheng, J. Xu, N. Matsuhisa, X. Yan, S. K. Kwon, M. Miyakawa, Z. Zhang, R. Ning, A. M. Foudeh, Y. Yun, C. Linder, J. B. H. Tok, Z. Bao, Strain-insensitive intrinsically stretchable transistors and circuits. *Nat. Electron.* **4**, 143–150 (2021).

50. T. Fu, S. Fu, J. Yao, Recent progress in bio-voltage memristors working with ultralow voltage of biological amplitude. *Nanoscale* **15**, 4669–4681 (2023).

51. T. Fu, X. Liu, H. Gao, J. E. Ward, X. Liu, B. Yin, Z. Wang, Y. Zhuo, D. J. F. Walker, J. J. Yang, J. Chen, D. R. Lovley, J. Yao, Bioinspired bio-voltage memristors. *Nat. Commun.* **11**, 1861 (2020).

52. S. Fu, H. Gao, S. Wang, X. Wang, T. Woodard, Z. Wang, J. Kong, D. R. Lovley, J. Yao, Constructing artificial neurons with functional parameters comprehensively matching biological values. *Nat. Commun.* **16**, 8599 (2025).

53. H. Jiang, Y. Sun, J. A. Rogers, Y. Huang, Mechanics of precisely controlled thin film buckling on elastomeric substrate. *Appl. Phys. Lett.* **90**, 133119 (2007).

54. Z. Yi, F. Xie, Y. Tian, N. Li, X. Dong, Y. Ma, Y. Huang, Y. Hu, X. Xu, D. Qu, X. Lang, Z. Xu, J. Liu, H. Zhang, B. Yang, A battery- and leadless heart-worn pacemaker strategy. *Adv. Funct. Mater.* **30**, 2000477 (2020).

55. L. Dong, A. B. Closson, M. Oglesby, D. Escobedo, X. Han, Y. Nie, S. Huang, M. D. Feldman, Z. Chen, J. X. J. Zhang, In vivo cardiac power generation enabled by an integrated helical piezoelectric pacemaker lead. *Nano Energy* **66**, 104085 (2019).

56. C. K. Jeong, J. H. Han, H. Palneedi, H. Park, G.-T. Hwang, B. Joung, S.-G. Kim, H. J. Shin, I.-S. Kang, J. Ryu, K. J. Lee, Comprehensive biocompatibility of nontoxic and high-output flexible energy harvester using lead-free piezoceramic thin film. *APL Mater.* **5**, 074102 (2017).

57. S. Azimi, A. Golabchi, A. Nekookar, S. Rabbani, M. H. Amiri, K. Asadi, M. M. Abolhasani, Self-powered cardiac pacemaker by piezoelectric polymer nanogenerator implant. *Nano Energy* **83**, 105781 (2021).

58. H. Zhang, X.-S. Zhang, X. Cheng, Y. Liu, M. Han, X. Xue, S. Wang, F. Yang, A. S. Smitha, H. Zhang, Z. Xu, A flexible and implantable piezoelectric generator harvesting energy from the pulsation of ascending aorta: In vitro and in vivo studies. *Nano Energy* **12**, 296–304 (2015).

59. X. Cheng, X. Xue, Y. Ma, M. Han, W. Zhang, Z. Xu, H. Zhang, H. Zhang, Implantable and self-powered blood pressure monitoring based on a piezoelectric thinfilm: Simulated, in vitro and in vivo studies. *Nano Energy* **22**, 453–460 (2016).

60. R. Yang, Y. Qin, C. Li, G. Zhu, Z. L. Wang, Converting biomechanical energy into electricity by a muscle-movement-driven nanogenerator. *Nano Lett.* **9**, 1201–1205 (2009).

61. J. Bae, J. Song, W. Jeong, K. R. Nandanapalli, N. Son, N. A. B. Zulkifli, G. Gwon, M. Kim, S. Yoo, H. Lee, H. Choi, S. Lee, H. Cheng, C. Kim, K. I. Jang, S. Lee, Multi-deformable piezoelectric energy nano-generator with high conversion efficiency for subtle body movements. *Nano Energy* **97**, 107223 (2022).

62. S. Lee, R. Hinchet, Y. Lee, Y. Yang, Z.-H. Lin, G. Ardila, L. Montès, M. Mouis, Z. L. Wang, Ultrathin nanogenerators as self-powered/active skin sensors for tracking eye ball motion. *Adv. Funct. Mater.* **24**, 1163–1168 (2014).

63. D. Wang, D. Zhang, P. Li, Z. Yang, Q. Mi, L. Yu, Electrospinning of flexible poly(vinyl alcohol)/MXene nanofiber-based humidity sensor self-powered by monolayer molybdenum diselenide piezoelectric nanogenerator. *Nano Micro Lett.* **13**, 57 (2021).

64. Y. Liu, L. Zhao, L. Wang, H. Zheng, D. Li, R. Avila, K. W. C. Lai, Z. Wang, Z. Xie, Y. Zi, X. Yu, Skin-integrated graphene-embedded lead zirconate titanate rubber for energy harvesting and mechanical sensing. *Adv. Mater. Technol.* **4**, 1900744 (2019).

65. W. Fan, R. Lei, H. Dou, Z. Wu, L. Lu, S. Wang, X. Liu, W. Chen, M. Rezakazemi, T. M. Aminabhavi, Y. Li, S. Ge, Sweat permeable and ultrahigh strength 3D PVDF piezoelectric nanoyarn fabric strain sensor. *Nat. Commun.* **15**, 3509 (2024).

66. J. Li, C. Carlos, H. Zhou, J. Sui, Y. Wang, Z. Silva-Pedraza, F. Yang, Y. Dong, Z. Zhang, T. A. Hacker, B. Liu, Y. Mao, X. Wang, Stretchable piezoelectric biocrystal thin films. *Nat. Commun.* **14**, 6562 (2023).

67. M. Han, H. Wang, Y. Yang, C. Liang, W. Bai, Z. Yan, H. Li, Y. Xue, X. Wang, B. Akar, H. Zhao, H. Luan, J. Lim, I. Kandela, G. A. Ameer, Y. Zhang, Y. Huang, J. A. Rogers, Three-dimensional piezoelectric polymer microsystems for vibrational energy harvesting, robotic interfaces and biomedical implants. *Nat. Electron.* **2**, 26–35 (2019).

68. X. Liu, H. Zhao, Y. Lu, S. Li, L. Lin, Y. Du, X. Wang, In vitro cardiomyocyte-driven biogenerator based on aligned piezoelectric nanofibers. *Nanoscale* **8**, 7278–7286 (2016).

69. E. Choi, S. Q. Lee, T. Y. Kim, H.-K. Chang, K. J. Lee, J. Park, MEMS-based power generation system using contractile force generated by self-organized cardiomyocytes. *Sens. Actuators B. Chem.* **151**, 291–296 (2010).

70. H. A. Sodano, D. J. Inman, G. Park, Comparison of piezoelectric energy harvesting devices for recharging batteries. *J. Intell. Mater. Syst. Struct.* **16**, 799–807 (2005).

71. A. Bayrashev, W. P. Robbins, B. Ziaie, Low frequency wireless powering of microsystems using piezoelectric–magnetostrictive laminate composites. *Sens. Actuators A. Phys.* **114**, 244–249 (2004).

72. G. L. Messing, S. Trolier-McKinstry, E. M. Sabolsky, C. Duran, S. Kwon, B. Brahmaroutu, P. Park, H. Yilmaz, P. W. Rehrig, K. B. Eitel, E. Suvaci, M. Seabaugh, K. S. Oh, Templated grain growth of textured piezoelectric ceramics. *Crit. Rev. Solid State Mater. Sci.* **29**, 45–96 (2004).
